# A new species of *Niceforonia* (Amphibia, Anura, Strabomantidae) from the Serranía de los Paraguas, Colombia, with the description of a new phenotypic synapomorphy for the *Niceforonia nigrovittata* species group

**DOI:** 10.3897/zookeys.1289.198411

**Published:** 2026-08-11

**Authors:** Jhon Jairo Ospina-Sarria, Andrés Felipe Rodríguez-Betancourt, Ximena Gómez-Sepúlveda, Iván Darío Morales-Vertel, Valentina Vélez-Franco, Nayely Gallego-Posada, Jose Daniel Herrera-Rengifo, John Edison Grajales Escobar, Irene Ceballos-Castro, Isabella García-Gómez, David Andrés Velásquez-Trujillo, Ayda Susana Ortiz Baez, Javier Mendez Narvaez

**Affiliations:** 1 Calima, Fundación para la Investigación de la Biodiversidad y Conservación en el Trópico, Cali, Colombia División Herpetología, Museo Argentino de Ciencias Naturales ‘Bernardino Rivadavia’ - CONICET Buenos Aires Argentina; 2 Unidad de Genómica Avanzada, Centro de Investigación y de Estudios Avanzados del Instituto Politécnico Nacional, Irapuato, México Departamento de Biodiversidad y Biología Experimental, Facultad de Ciencias Exactas y Naturales, Universidad de Buenos Aires Buenos Aires Argentina; 3 Grupo de Investigación Biodiversidad Unicordoba, Departamento de Biología, Facultad de Ciencias Básicas Universidad de Córdoba, Montería, Colombia Engineering Faculty, Universidad Central del Valle del Cauca Tuluá Colombia; 4 Departamento de Ciencias Naturales y Matemáticas, Pontificia Universidad Javeriana, Cali, Colombia Departamento de Biología, Facultad de Ciencias Básicas y Tecnologías, Universidad del Quindío Armenia Colombia; 5 Departamento de Biología, Facultad de Ciencias Básicas y Tecnologías, Universidad del Quindío, Armenia, Colombia Biogeography Department, Trier University Trier Germany; 6 Corporación Serraniagua, El Cairo, Valle del Cauca, Colombia School of Medical Sciences, University of Sydney Sydney Australia; 7 Propacífico, Asociación de Productores Agroecológicos de la Vereda El Pacífico, El Cairo, Valle del Cauca, Colombia Calima, Fundación para la Investigación de la Biodiversidad y Conservación en el Trópico Cali Colombia; 8 Grupo Ambiental BioBalsal, El Balsal, Versalles, Valle del Cauca, Colombia Grupo de Investigación Biodiversidad Unicordoba, Departamento de Biología, Facultad de Ciencias Básicas Universidad de Córdoba Montería Colombia; 9 División Herpetología, Museo Argentino de Ciencias Naturales ‘Bernardino Rivadavia’ - CONICET, Buenos Aires, C1405DJR, Argentina Unidad de Genómica Avanzada, Centro de Investigación y de Estudios Avanzados del Instituto Politécnico Nacional Irapuato Mexico; 10 Departamento de Biodiversidad y Biología Experimental, Facultad de Ciencias Exactas y Naturales, Universidad de Buenos Aires, Buenos Aires, Argentina Departamento de Ciencias Naturales y Matemáticas, Pontificia Universidad Javeriana Cali Colombia; 11 Laboratorio de Genética Evolutiva, Instituto de Biología Subtropical-CONICET, Posadas, Misiones, Argentina Corporación Serraniagua El Cairo Colombia; 12 Biogeography Department, Trier University, 54286 Trier, Germany Propacífico, Asociación de Productores Agroecológicos de la Vereda El Pacífico El Cairo Colombia; 13 School of Medical Sciences, University of Sydney, Sydney, New South Wales 2006, Australia Grupo Ambiental BioBalsal Versalles Colombia; 14 Engineering Faculty, Universidad Central del Valle del Cauca, Tuluá, Colombia Laboratorio de Genética Evolutiva, Instituto de Biología Subtropical-CONICET Posadas Argentina

**Keywords:** Brachycephaloid frogs, Cerro El Inglés, evolution, Key Biodiversity Area (KBA), taxonomy

## Abstract

A new species of *Niceforonia* is described from the montane cloud forests of southwestern Colombia. The new species is distinguished from all congeners by possessing dorsolateral folds, a visible tympanic membrane, a fleshy lip swelling in adult males, nuptial pads, circumferential grooves on all fingers, a reddish-brown to grayish dorsal coloration, yellow ventral and hidden surfaces, and dark supra-inguinal spots. Molecularly, the new species differs from all congeners by an uncorrected *p*-distance of > 7% for the 16S rRNA gene fragment. Furthermore, the possession of dark supra-inguinal spots optimizes unambiguously as a synapomorphy for two clades within Strabomantidae. The first clade comprises members of the genus *Niceforonia* and includes *N. brunnea*, *N. dracula*, *N. elassodiscus*, *N. mantipus*, *N. nigrovittata*, *N. quilloturo*, *N. peraccai*, four candidate species, and the new species described herein. This clade is referred to as the *Niceforonia nigrovittata* species group, diagnosed by the presence of dark supra-inguinal spots. By retaining this name, we ensure taxonomic stability and honor the well-established historical use of the nomenclature in literature. The second clade comprises members of the subfamily Holoadeninae, including the genera *Bahius*, *Barycholos*, *Phyllonastes*, and *Urkuphryne*. A previous study already proposed a phenotypic synapomorphy for the group composed of *Phyllonastes* + *Barycholos*. Consequently, the available evidence corroborates the monophyly of both the group comprising these four holoadenine genera and the *Phyllonastes* + *Barycholos* group. This congruence provides a robust, nested phylogenetic resolution and precise taxonomic diagnoses, demonstrating how the integration of molecular and phenotypic evidence is a powerful tool for testing evolutionary hypotheses in brachycephaloid frogs.

## Introduction

Among the direct-developing frogs of the superfamily Brachycephaloidea, many species have enlarged discs on the tips of their digits (Lynch 1989; Lynch and Duellman 1997; Duellman and Lehr 2009). Accordingly, the uncommon occurrence of narrow discs on the digits has been used to define infrageneric units within the superfamily Brachycephaloidea (e.g., Lynch 1975, 1976). One of these infrageneric units was the *Eleutherodactylus discoidalis* species group, which included species distributed over the Amazonian Andean versant in Bolivia, southern Peru, northern Ecuador, and Andean foothills in northern Argentina (Lynch 1976). It was not until the late 80’s that Lynch (1989) separated the *E. discoidalis* species group into three infrageneric units, *E. discoidalis*, *E. dolops*, and *E. nigrovittatus* species groups.

Decades later, the inclusion of molecular data in phylogenetic analyses corroborated the proposal of Lynch (1989) and refined the systematics of these groups. For example, Padial et al. (2008) demonstrated that species of the *E. discoidalis* group belong to the genus *Oreobates*, whereas Hedges et al. (2008a) demonstrated that species of the *E. dolops* and *E. nigrovittatus* groups (see also Lynch and Duellman 1997; Duellman and Pramuk 1999; Lehr 2005), with species formerly placed in *Phrynopus*, form a monophyletic group distinct from both *Eleutherodactylus* and *Phrynopus*. Consequently, the genus *Isodactylus* Hedges, Duellman & Heinicke, 2008a was proposed to accommodate them. The generic name *Isodactylus* was found to be a junior homonym and was subsequently replaced with *Hypodactylus* by Hedges et al. (2008b). This replacement name derives from the Greek *hypo* (less than) and *daktylos* (toe), referring to the narrow digital discs that are characteristic of the genus (Hedges et al. 2008b).

A decade later, the systematic arrangement of the genus was further modified when additional lineages were evaluated. Acosta-Galvis et al. (2018) demonstrated that *Niceforonia nana*Goin & Cochran, 1963 is nested within *Hypodactylus*; consequently, the genus *Hypodactylus* was synonymized with *Niceforonia*. Currently, *Niceforonia* is diagnosed solely through molecular data. Therefore, when molecular data are missing, species are assigned to the genus based on overall morphological similarity, with narrow digital discs serving as a primary diagnostic character. However, Ospina-Sarria and Grant (2022) demonstrated that the traditional coding of digital discs in Brachycephaloidea conflates several independent characters, including the presence of discs on digits, their shape, and their degree of lateral expansion (e.g., narrow vs broad digital discs), which in turn can vary independently across different digits. Consequently, until the variation obscured by this traditional coding system is evaluated within a framework of transformational independence, its phylogenetic significance will remain unknown.

More recently, Reyes-Puig et al. (2026) described two *Niceforonia* species from Ecuador and highlighted the existence of additional undescribed taxa. The genus is currently composed of 17 species distributed from the three Colombian Cordilleras southwards through Ecuador to the Cordillera Oriental in central Peru, and eastward into the Amazonian Basin of Ecuador and northern Peru. Given the reliance on molecular data for diagnosing *Niceforonia* and the limited understanding of its phenotypic evolution, we herein investigate the phylogenetic significance of dark supra-inguinal spots, a character first reported in *N. nigrovittata* (Andersson, 1945) and *N. mantipus* (Boulenger, 1908) by Lynch and Duellman (1980) and Lynch (1989). This analysis aims to evaluate the phylogenetic significance of dark supra-inguinal spots in *Niceforonia* and related genera. Additionally, we included samples of *Niceforonia mantipus* from several localities (including the type locality) in our analysis and describe a new species of *Niceforonia* from the Serranía de los Paraguas, Colombia.

## Materials and methods

### Field surveys and specimen collection

**Figure 1.**
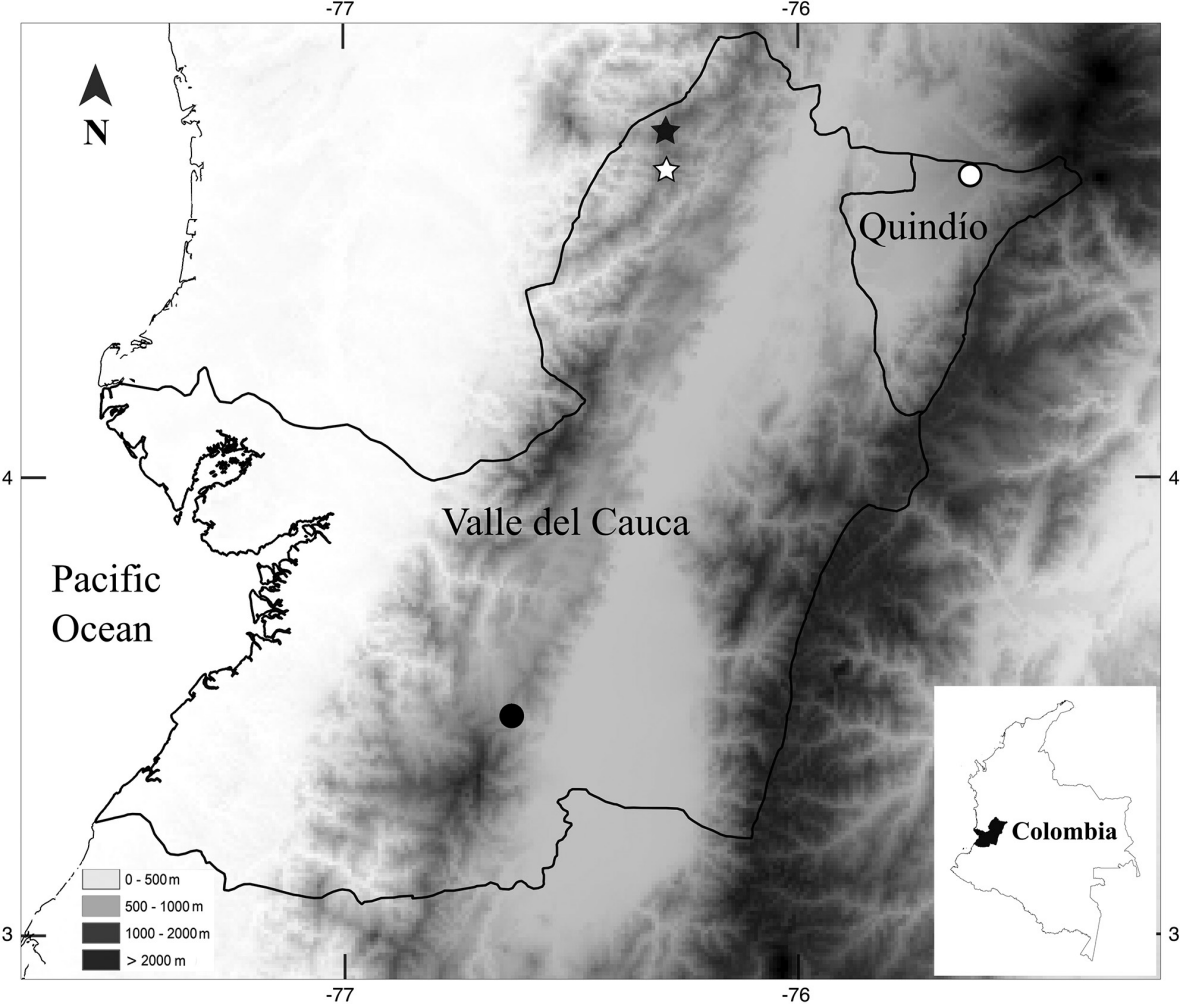
Map of southwestern Colombia (inset) showing the distribution of *Niceforonia mantipus* (circles) and *Niceforonia tempestuosa* (stars). Black symbols represent the type localities of *N. mantipus* (Bosque de San Antonio) and *Niceforonia tempestuosa* (Cerro El Inglés) in Valle del Cauca. White circle indicates additional record for *N. mantipus* in Reserva Natural Bremen (Filandia, Quindío). The white star represents the record for *Niceforonia tempestuosa* in El Mirador (Balsal, Versalles, Valle del Cauca).

We visited two sites in the Cordillera Occidental of Colombia, the Reserva Natural Comunitaria Cerro El Inglés (4°45'N, 76°17'W) during two fieldwork expeditions (18–28 October 2024 and 18–28 April 2025) and the Bosque de San Antonio (also known as Cerro de la Horqueta, 3°29'N, 76°37'W) during a fieldwork expedition (11 November 2024) (Fig. 1). These surveys were carried out under the framework of our long-term project for the study and conservation of amphibian diversity from the Key Biodiversity Area (KBA) Serranía de los Paraguas. The Reserva Natural Comunitaria Cerro El Inglés is a cloud forest ecosystem in the Serranía de los Paraguas KBA between the western Andes and the biogeographical Chocó with an approximate extension of 1000 hectares located in the municipality El Cairo, Department of Valle del Cauca, Colombia. The Bosque de San Antonio is a montane summit within the San Antonio Forest/Km 18 KBA in the Cordillera Occidental covering ca 800 hectares, located 15 km west of Cali along the road to Buenaventura, Colombia.

A team of ten researchers conducted the fieldwork. Diurnal searches were performed from 10:00 to 14:00 h, and nocturnal surveys from 20:00 to 02:00 h. We performed visual encounter surveys in riparian zones, forest edges, and interiors, where leaf-litter shifting was employed to locate cryptic or leaf-dwelling individuals. Upon encounter, frogs were captured by hand in plastic bags, their geographical coordinates were recorded with a Garmin Global Positioning System (GPS) unit, and individuals were immediately transferred to a clear area for photographic documentation with a Nikon D90 camera. Subsequently, upon the team’s return to the base camp, the frogs were euthanized with an intraperitoneal injection of Xylocaine (lidocaine hydrochloride). Tissue samples (a piece of muscle) were removed and stored in 95% ethanol for molecular analyses, before preservation of the specimens. Specimens were individually tagged and fixed in 10% formalin. Upon returning to the laboratory, specimens were rinsed in several changes of water and finally transferred to 70% ethanol for long-term storage. All specimens were deposited in the Colección de Anfibios y Reptiles de la Universidad del Quindío, Armenia, Colombia (**ARUQ**).

### Morphology

Terminology for phenotypic characters and standardized format for definitions follows Lynch and Duellman (1980, 1997) and Duellman and Lehr (2009). To estimate lengths of toes III and V, we adpressed both toes against toe IV, and for lengths of fingers I and II, we appressed those fingers against each other. Maturity in males was judged by the presence of enlarged testes and secondary sexual characters (vocal slits and non-spinous nuptial pads) while maturity in females was assumed if the oviducts were large and convoluted and/or if enlarged ova were evident. Color pattern in life was taken from direct observation of the specimens after capture and high definition color digital photographs. The following standard abbreviations are used for convenience: **SVL** (snout-to-vent length), **HW** (head width), **E-N** (eye-nostril distance), and **IOD** (interorbital distance). Measurements were made using digital calipers to the nearest 0.1 mm, under a Carl Zeiss Stemi 2000-C stereoscopic microscope. Institutional abbreviations are: **AMNH** (American Museum of Natural History, Division of Vertebrate Zoology, New York 10024, USA), **ARUQ** (Colección de Anfibios y Reptiles de la Universidad del Quindío, Armenia, Colombia), **ICN** (Instituto de Ciencias Naturales, Museo de Historia Natural, Universidad Nacional de Colombia, Bogotá), **KU** (Biodiversity Institute, University of Kansas), **MHN-UIS** (Museo de Historia Natural, Universidad Industrial de Santander, Bucaramanga, Colombia), **USNM** (National Museum of Natural History, Division of Amphibians and Reptiles, Washington), and **UTA** (University of Texas at Arlington, Department of Biological Sciences, Arlington). All specimens examined are listed in Suppl. material 1.

### Molecular phylogenetic analyses

We conducted a phylogenetic analysis of DNA sequences from two mitochondrial (12S and 16S, 2866 bp) and two nuclear (recombinase activation 1 RAG-1, 631 bp, and tyrosinase TYR, 531 bp) genes. Sequences were obtained from GenBank or sequenced for this study. Total genomic DNA was extracted from ethanol-preserved muscle tissue using the GeneJET Genomic DNA Purification kit (Thermo Fisher Scientific, Inc.) according to the manufacturer’s protocol. PCR amplification was carried out following the protocols of Santos et al. (2003). The mitochondrial gene 16S was amplified using the primers 16SCL-16SDH (Santos et al. 2003). Purified PCR products were sequenced in a single direction using an automated sequencer (Macrogen Inc., South Korea). The resulting chromatograms were automatically evaluated using the quality-flagging features in Sequencher v.5.0.1 (Gene Codes, Ann Arbor, MI, USA). Low-confidence positions, ambiguous peaks, and low-quality terminal regions at both ends were visually inspected and manually trimmed before alignment. Complete sequences were edited with the software GENEIOUS PRO v.6.1.6 (Kearse et al. 2012).

For the phylogenetic analyses, we included representative sequences of all known species of *Niceforonia* for which sequences are available in GenBank (i.e., *N. brunnea* Lynch, 1975, *N. dracula*Reyes-Puig et al. 2026, *N. elassodiscus* Lynch, 1973, *N. nana*, *N. nigrovittata*, *N. quilloturo* Reyes-Puig et al., 2026, *N. peraccai* Lynch, 1975, and *N. philippi* Jiménez de la Espada, 1875). Furthermore, our dataset incorporates the four candidate species and the complex of *N. nigrovittata* reported by Reyes-Puig et al. (2026). Additionally, we provided new molecular data for *Niceforonia mantipus* and the new species described herein. We followed the family classification of Hedges et al. (2008a) and Heinicke et al. (2018). Outgroup sampling was designed to test the monophyly of *Niceforonia* and the polarity of morphological character states, including all genera recognized within Strabomantidae (a total of 198 terminals were included in the analysis). A species of the family Craugastoridae (*Craugastor longirostris* Boulenger, 1898) was included to root the trees. GenBank accession numbers and voucher specimens for all taxa and molecular markers used in this study are listed in Suppl. material 2.

Sequences were aligned using the online server of MAFFT software v.7 (http://mafft.cbrc.jp/alignment/server/large.html) (Katoh and Toh 2008; Katoh et al. 2019) under the strategy E-INS-i (for the 12S and 16S fragments) and G-INS-i (the remaining fragments) with default parameters for gap opening and extension. Phylogenetic relationships were inferred using Maximum Likelihood (ML) and Parsimony approaches. ML analyses were conducted in IQ-TREE v.3.1.1 (Wong et al. 2025), employing ModelFinder (Kalyaanamoorthy et al. 2017) to determine the optimal partition scheme and substitution models. The best partition scheme included three subsets (see Table 1). Nodal support was assessed via 1,000 ultrafast bootstrap (UFBoot) replicates (Hoang et al. 2018), incorporating a nearest-neighbor interchange search (-bnni) to mitigate the overestimation of branch support. The parsimony analysis was performed in TNT v.1.6 (Goloboff et al. 2008; Goloboff and Morales 2023), using the New Technology Search algorithms. Gaps were considered as a fifth state and transformation events were equally weighted. Parsimony jackknife support (absolute frequencies) was calculated with 1,000 replicates, each employing 50 random addition sequences and TBR branch swapping. These support values were mapped onto the Most Parsimonious Trees obtained from an initial driven search of 100 hits using the New Technology search algorithms of Nixon (1999) and Goloboff (1999). We favored parsimony as the optimality criterion (gaps treated as a fifth state), as this minimizes the number of required transformations and generates the most parsimonious hypotheses, which are more readily subject to falsification (Kluge 2005; Kluge and Grant 2006). The resulting trees were visualized and edited through the iTOL phylogenetic tree viewer (Letunic and Bork 2007) and INKSCAPE (XQuartz v.2.8.1, http://www.inkscape.org/).

**Table 1.** Best partition scheme and best-fit models selected by ModelFinder for the IQ-TREE analysis.

**Subset**	**Data blocks**	**Model**
1	Nuclear coding: Rag1 (1^st^, 2^nd^) + Tyr (2^nd^, 3^rd^)	TPM3u+F+I+R3
2	Nuclear coding: Rag1 (3^rd^) + Tyr (1^st^)	TIM+R3
3	Non-coding mitochondrial (12S, 16S, Val-tRNA)	GTR+F+R8

Uncorrected *p*-distances of the 16S gene sequences were calculated using the software PAUP v.4.0 (Swofford 2002) for a dataset with all sequences having the same length and no missing data (472 bp including gaps) and containing only sequences from genus *Niceforonia*. Due to their limited length (< 263 bp), *Niceforonia nana*, one specimen of *N. nigrovittata* (CORBIDI9547), one of *N. peraccai* (QCAZA57536), and one of *Niceforonia* sp 3 (QCAZA68992) were excluded from the *p*-distance matrix to avoid biased estimates resulting from insufficient overlap. The sequences were aligned using the software MAFFT in GENEIOUS PRO v.6.1.6 under the strategy G-INS-i with default parameters for gap opening and extension.

### Evaluation of diagnostic character states

We evaluated the distribution of dark supra-inguinal spots across *Niceforonia* and related genera using evidence collected from museum specimens, literature reviews, and field observations. The supra-inguinal region is located on the posterior flank, immediately dorsal to the groin. Although the dark supra-inguinal spots may merge with the dark dorsolateral pattern, they should be treated as independent characters, as their distributions are not coextensive. This character was originally reported by Lynch and Duellman (1980) and Lynch (1989) for *N. nigrovittata* and *N. mantipus*. Details regarding the taxonomic distribution of this character and their respective sources are provided in Suppl. material 1. We used YBYRÁ (Machado 2015) and TNT v.1.6 (Goloboff and Morales 2023) to identify and plot unambiguously optimized synapomorphies shared across all optimal trees. YBYRÁ generates color-coded boxes to indicate if a derived state occurs only in the clade in question (non-homoplastic) or also occurs in other clades (homoplastic) and if it is shared by all terminals of the clade (unique) or is subsequently transformed into one. The phylogenetic significance of dark supra-inguinal spots was also discussed in the context of the systematics proposed by Portik et al. (2023) and Ortega et al. (2025).

**Figure 2.**
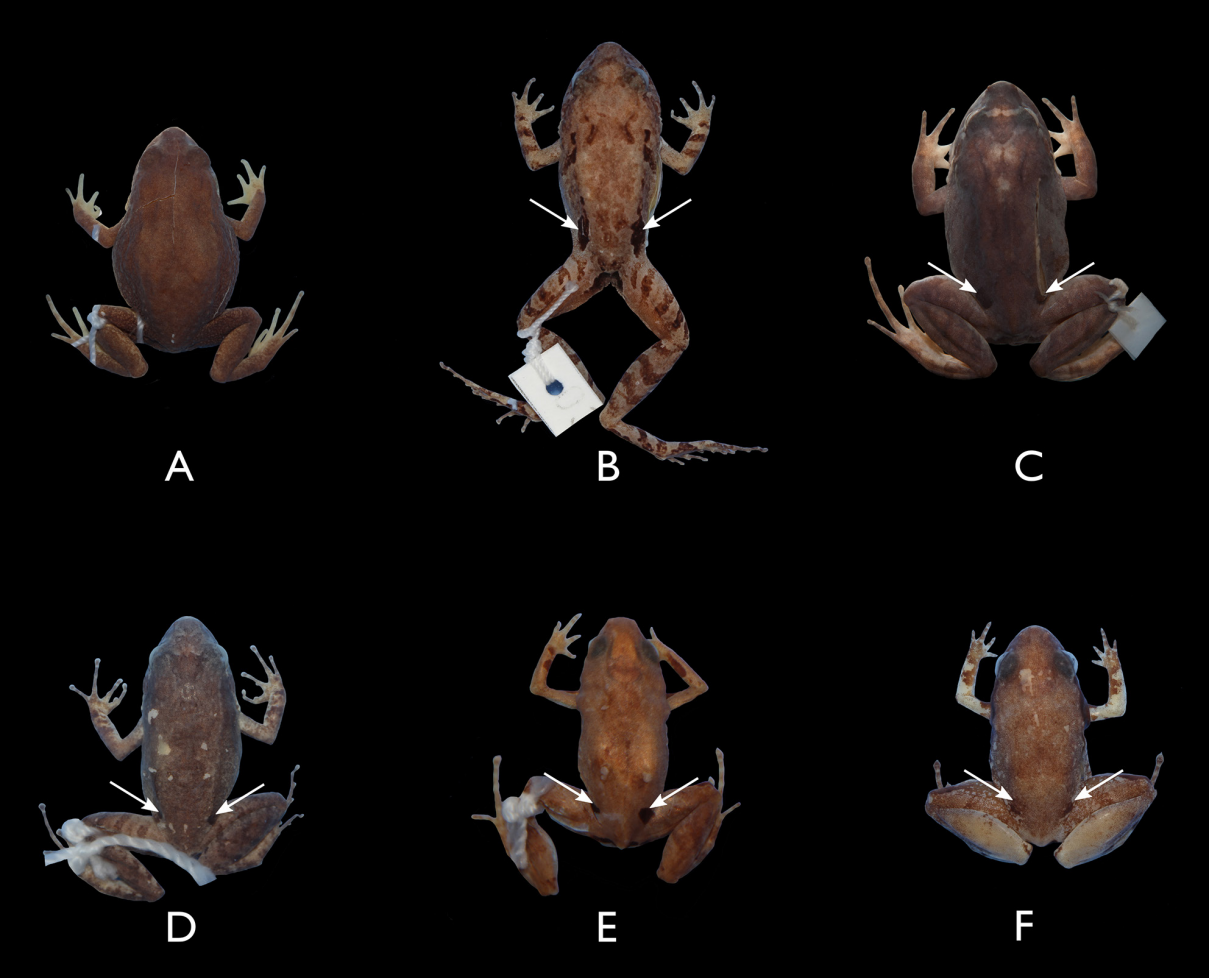
Dorsal view showing the variation of dark supra-inguinal spots (white arrows) across representative species of the family Strabomantidae. **A**. *Niceforonia nana* (KU 169125, SVL 19.2 mm, dark supra-inguinal spots absent); **B**. *N. nigrovittata* (KU 123546, SVL 25.0 mm); **C**. *N. elassodiscus* (ICN 49666, SVL not available); **D**. *Barycholos pulcher* (KU 146044, SVL 21.0 mm); **E**. *Phyllonastes lochites* (KU 147070, SVL 14.9 mm); **F**. *Phyllonastes myrmecoides* (KU 220578, SVL 11.0 mm).

The definition of the morphological character and its states is as follows: occurrence of dark supra-inguinal spots: state 0, absent; state 1, present (Fig. 2).

## Results

### Molecular phylogeny

**Figure 3.**
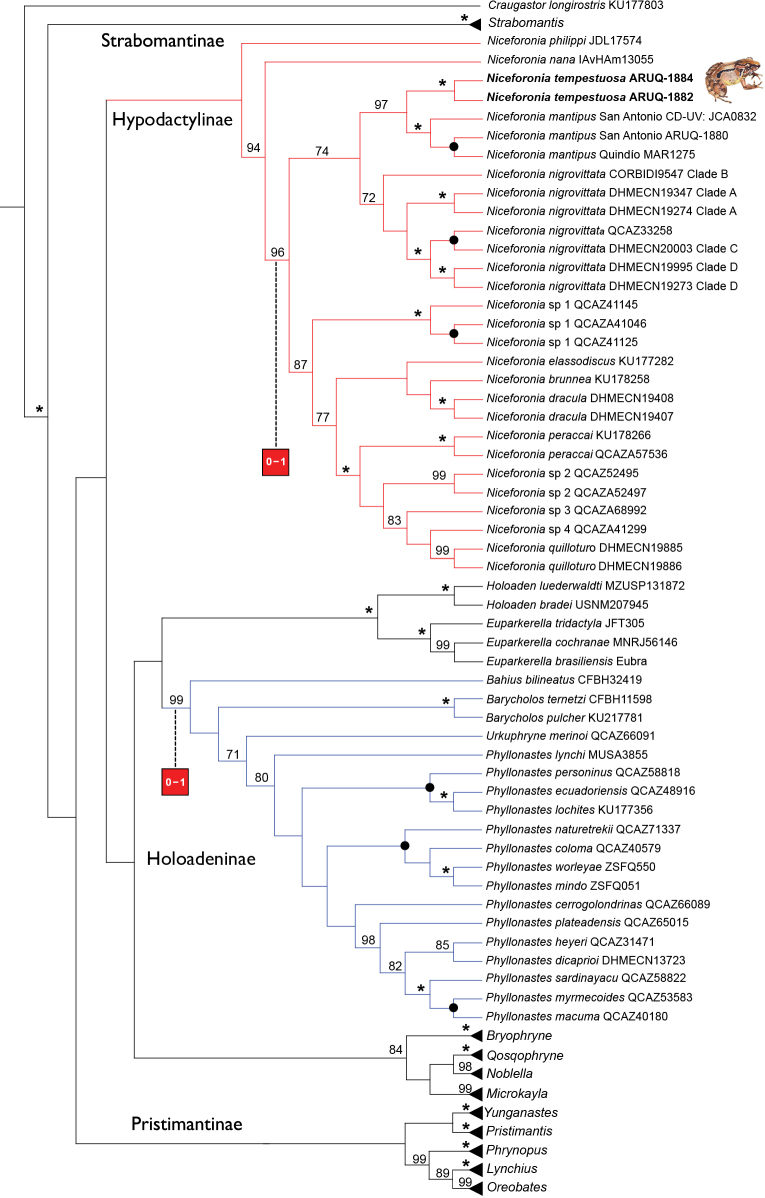
Phylogenetic relationships of *Niceforonia* and outgroups recovered in one of the 97 most-parsimonious trees of 24,174 steps from a parsimony analysis in TNT treating gaps as a fifth state. Some genera are shown as collapsed clades; the complete tree is provided in Suppl. material 3. Black dot indicates nodes that collapse in the strict consensus. Numbers on nodes are Parsimony jackknife absolute frequencies values. Nodes without values indicate a value < 70% of the jackknife value and an asterisk indicates a 100%. Character states with primitive-derived states are indicated within the squares (see text and Suppl. material 1 for character definitions). Color-coding: red and blue highlight the genus *Niceforonia* and a clade comprising of *Bahius*, *Barycholos*, *Phyllonastes*, and *Urkuphryne*, respectively; red squares indicate unique, homoplastic.

The phylogenetic analysis using parsimony as optimality criterion (gaps treated as a fifth state) resulted in 97 most-parsimonious trees of 24,174 steps. One of these trees is shown in Fig. 3. The conflict between these optimal trees involves relationships among congeners of *Bryophryne*, *Lynchius*, *Microkayla*, *Noblella*, *Phyllonastes* as well as among conspecific specimens of *Niceforonia mantipus* and *N. nigrovittata*. Our molecular phylogeny recovered *Niceforonia* as a monophyletic group with low support (Jackknife < 56%). Similarly, its sister-group relationship with Holoadeninae received only low support (Jackknife = 11%). The relationship between *Niceforonia* (Hypodactylinae) + Holoadeninae, and the subfamily Pristimantinae showed low support (Jackknife = 20%). Furthermore, a highly supported sister-group relationship (Jackknife = 100%) was recovered between Strabomantinae and the clade composed of Hypodactylinae, Holoadeninae, and Pristimantinae.

Comparison between parsimony and ML topologies revealed topological conflicts among species within the same genus; however, deeper nodes representing intergeneric relationships were robustly recovered and congruent across both analyses (Fig. 3; Suppl. materials 3, 4). The only exceptions to the intergeneric congruence involved the placement of *Niceforonia* (Hypodactylinae), *Barycholos* and the relationships within the *Qosqophryne*–*Noblella*–*Microkayla* clade. Notably, the positions of all these clades were poorly supported (Jackknife < 70%; UFB < 95%). Under parsimony, *Niceforonia* was recovered as sister to Holoadeninae, whereas Maximum Likelihood (ML) placed it as sister to Pristimantinae. Conversely, *Barycholos* was recovered as sister to the *Urkuphryne* + *Phyllonastes* clade in the parsimony analysis, whereas Maximum Likelihood (ML) placed it as sister to a broader clade including *Bahius*, *Urkuphryne*, and *Phyllonastes*. Finally, the position of *Microkayla* shifted from being sister to *Qosqophryne* + *Noblella* in the parsimony analysis to being recovered as sister to *Qosqophryne* (with this pair being sister to *Noblella*) in the ML tree (Fig. 3; Suppl. materials 3, 4).

Phylogenetic relationships within *Niceforonia* were congruent across both analytical approaches (Fig. 3; Suppl. materials 3, 4), with the sole exception of the relative positions of *N. dracula*, *N. brunnea*, and *N. elassodiscus*. Under parsimony, *N. dracula* is sister to *N. brunnea*, which are in turn sister to *N. elassodiscus*. In contrast, in the Maximum Likelihood (ML) analysis, *N. dracula* is sister to *N. elassodiscus*, and this clade is sister to *N. brunnea*. In both analyses, the new species was recovered as sister to *N. mantipus*. The uncorrected *p*-distances for the 16S gene showed a relatively high genetic differentiation among species of the genus *Niceforonia* (Table 2). On the basis of phenotypic and molecular evidence, below we provide a formal description of a new species of *Niceforonia* from the Serranía de los Paraguas, Colombia.

**Table 2.** Percentage of uncorrected *p*-distances of the 16S gene of *Niceforonia* species. *Niceforonia tempestuosa* is shown in bold.

**Taxon (n)**	**1**	**2**	**3**	**4**	**5**	**6**	**7**	**8**	**9**	**10**	**11**	**12**	**13**	**14**
1 *N. brunnea*	0.00													
2 *N. dracula* (2)	6.07–6.29	0.00												
3 *N. elassodiscus*	5.84	5.65–5.86	0.00											
4 *N. mantipus* (3)	10.54	10.34	11.01	0.00										
5 *N. nigrovittata* Clade A (2)	11.01	12.14–12.35	13.20	12.29	0.00									
6 *N. nigrovittata* Clade C (2)	8.02–8.20	9.19–9.38	8.49–8.67	9.2–9.37	9.52–9.58	0.00								
7 *N. nigrovittata* Clade D (2)	8.41–8.45	9.13–9.17	8.43–8.46	10.03–10.07	9.72–9.75	1.33–1.78	0.00							
8 *N. quilloturo* (2)	6.07–6.51	6.53–6.75	5.65–5.86	11.91–12.35	12.79–13.23	10.34–10.94	10.30–10.71	0.00						
9 *N. peraccai*	5.42	6.53–6.75	5.20	10.13	11.71	8.96–9.14	8.68–8.71	4.12–4.56	0.00					
10 *N. philippi*	17.60	16.79–17.01	17.36	18.12	17.36	16.67–16.75	15.98–16.04	17.62–18.06	17.01	0.00				
11 *N.* sp 1 (3)	5.46–5.83	6.52–6.94	7.35–7.36	10.30–10.33	12.12–12.31	8.88–9.13	9.09–9.29	7.15–7.59	6.71–6.73	17.83–17.85	0.00			
12 *N.* sp 2 (2)	6.49–8.00	7.61–9.57	5.85–7.58	11.90–13.21	14.06–14.71	10.04–10.99	9.81–10.55	4.55–6.06	4.11–5.85	17.18–18.05	7.79–9.10	0.00		
13 *N.* sp 4	5.85	6.10–6.31	6.28	11.92	6.50	10.56–10.72	10.48–10.52	1.30–1.73	4.78	16.97	6.50	3.90–5.20	0.00	
**14 *N. tempestuosa* (2)**	**12.52–12.55**	**13.86–13.90**	**13.22–13.25**	**7.44–7.45**	**13.62–13.64**	**10.00–10.08**	**10.00–10.05**	**14.78–15.25**	**12.79–12.82**	**18.29–18.33**	**12.50–12.56**	**14.08–15.01**	**14.78–14.81**	**0.00**

### Systematics

#### Family: Strabomantidae Hedges, Duellman & Heinicke, 2008a

**Genus: *Niceforonia*Goin & Cochran, 1963**

##### Niceforonia tempestuosa sp. nov.

Taxon classificationAnimaliaAnuraStrabomantidae

0B85AE96-8721-5775-A880-D63DF730612D

https://zoobank.org/D5331657-A4E6-4A86-992D-ABCD98FB6A78

**Figure 4.**
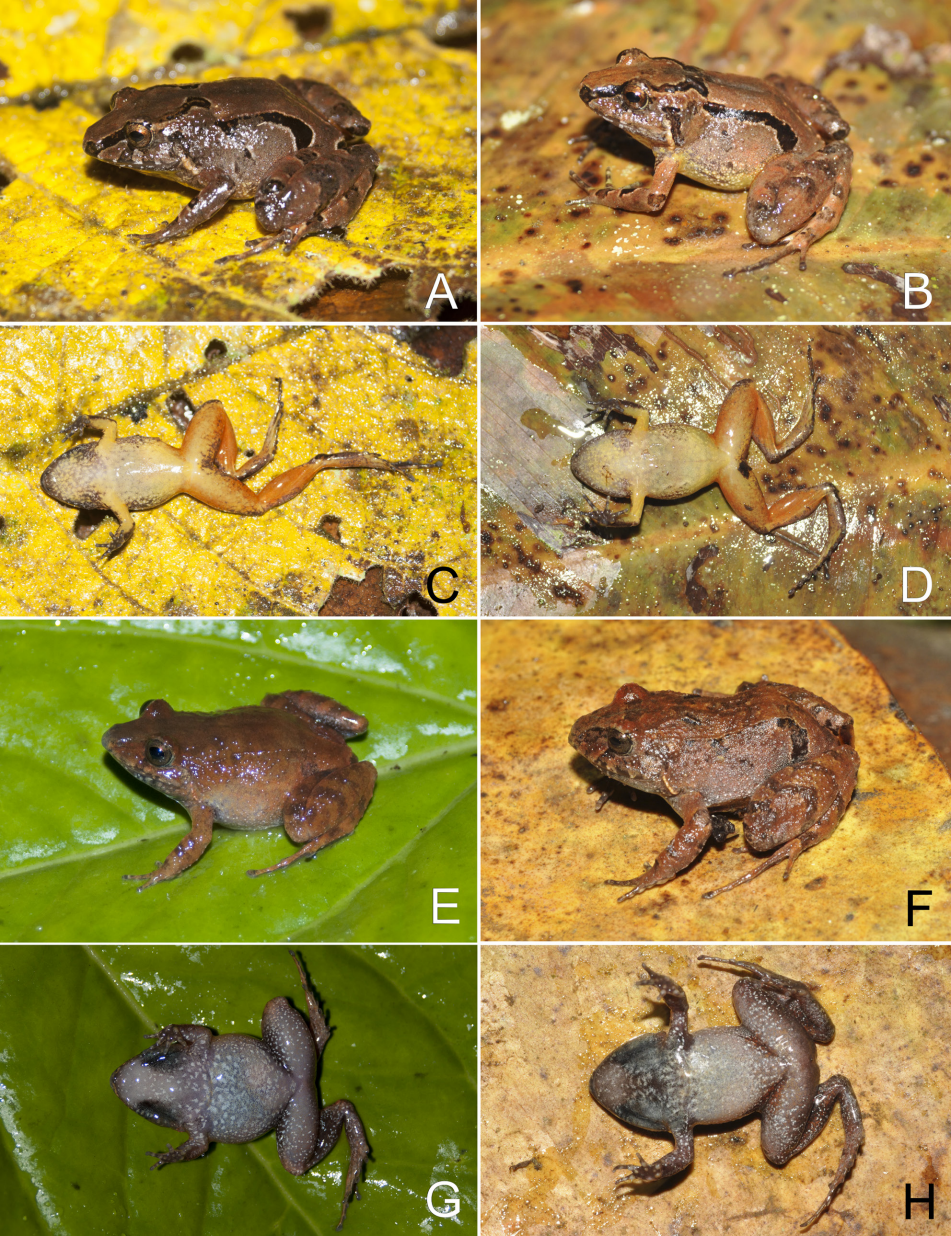
Living specimens of *Niceforonia tempestuosa* and *N. mantipus*. Dorsal and ventral views of adult males of *Niceforonia tempestuosa*. **A, C**. ARUQ-1885, SVL 24.5 mm; **B, D**. ARUQ-1882, SVL 23.8 mm and *N. mantipus*; **E, G**. Specimen not collected; **F, H**. ARUQ-1881, SVL 23.5 mm.

**Figure 5.**
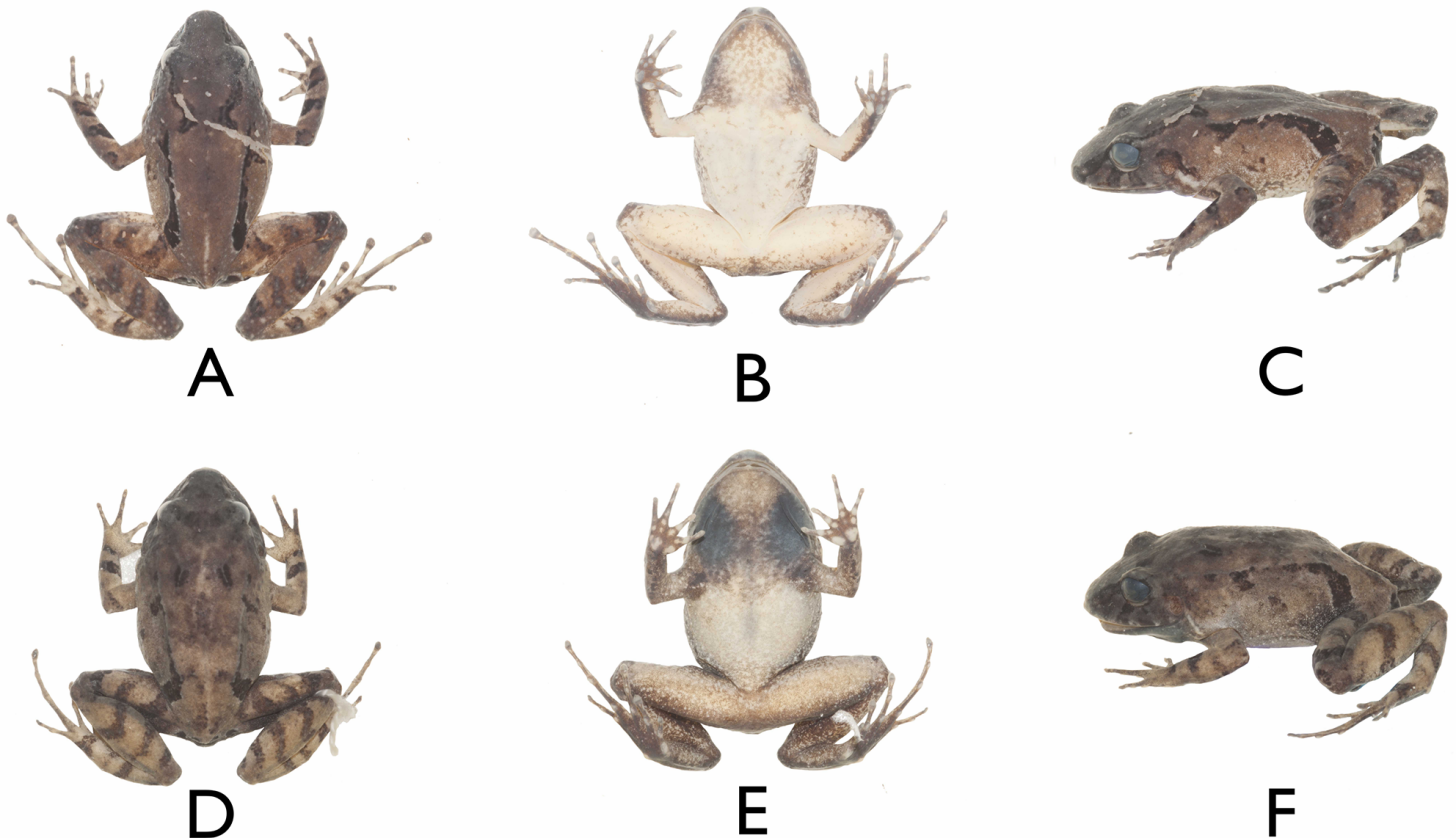
Dorsal, ventral, and lateral views of *Niceforonia tempestuosa*. **A–C**. Holotype (ARUQ-1885, SVL 24.5 mm); **D–F**. *Niceforonia mantipus* (ARUQ-1881, SVL 23.5 mm).

**Figure 6.**
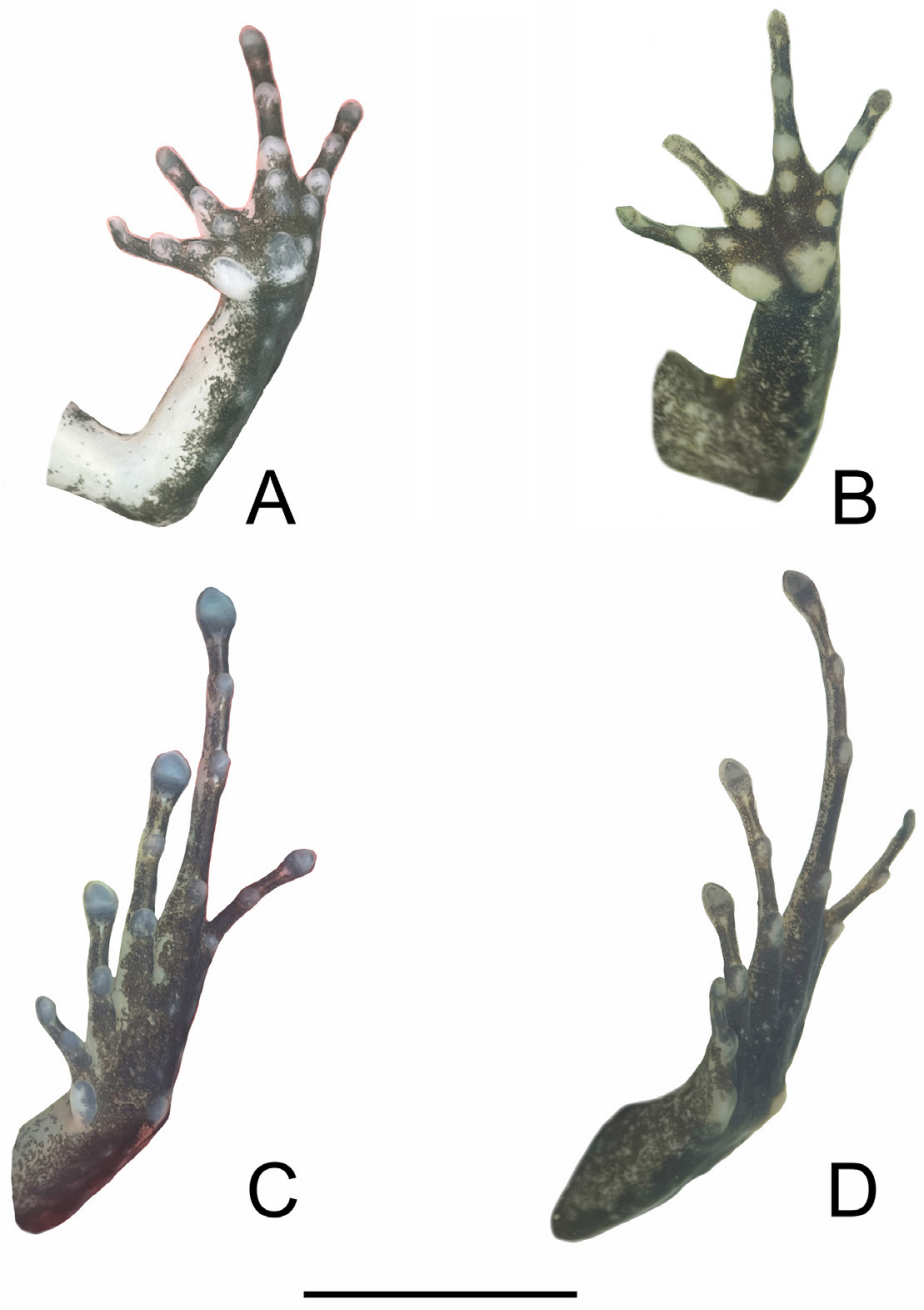
Ventral views of the hand and foot of preserved adult male specimens of *Niceforonia tempestuosa* and *N. mantipus*. **A, C**. Hand and foot of *Niceforonia tempestuosa* (ARUQ-1883); **B, D**. *N. mantipus* (ARUQ-1881). Scale bar: 6 mm.

Figs 4A–D, 5A–C, 6A, 6C

###### Proposed standard Spanish name.

Rana de los bosques montanos nublados.

###### Proposed standard English name.

Montane cloud forest frog.

###### Type material.

***Holotype***. • ARUQ-1885 (field no. JJS 702), an adult male from an interior forest at a site named “Corazón” within the Reserva Natural Comunitaria Cerro El Inglés (4°45'N, 76°17'W, 2261 m), Municipality of El Cairo, Valle del Cauca, Colombia; collected by Jhon Jairo Ospina-Sarria, Andrés Felipe Rodríguez-Betancourt, Ximena Gómez-Sepúlveda, Iván Darío Morales-Vertel, Valentina Vélez-Franco, Irene Ceballos-Castro, and Isabella García-Gómez on 25 October 2024. ***Paratypes***. A total of 11 specimens. • ARUQ-1882 (adult male) and ARUQ-1883 (juvenile male), collected on 24 October 2024; • ARUQ-1884 (adult male), collected with the holotype on 25 October 2024; • ARUQ-1889, 1891, 1893 (adult males), ARUQ-1892 (subadult male), ARUQ-1890 (subadult female), and ARUQ-1886 (juvenile male), collected on 25 April 2025; all from Corazón, Reserva Natural Comunitaria Cerro El Inglés. • ARUQ-1887 (adult female) and ARUQ-1888 (subadult female), collected on 20 April 2025 from Bosque Puente Quebrada Las Amarillas, Reserva Natural Comunitaria Cerro El Inglés.

###### Diagnosis.

*Niceforonia tempestuosa* is diagnosed by the following combination of characters: (1) skin of dorsum smooth with scattered small warts; dorsolateral and \ /-shaped scapular folds present; venter smooth, discoidal fold absent; (2) tympanic membrane differentiated, upright-oval to round; its length 33.3**–**44.8% of eye length in six adult males and 47% in one adult female; prominent tympanic annulus, its upper edge covered by supratympanic fold, extending only just posterior to tympanic annulus; (3) snout subacuminate in dorsal view, rounded in profile; a fleshy swelling of lip in adult males; canthus rostralis straight; (4) upper eyelid bearing minute tubercles, narrower than IOD (62.0–85.1% IOD); interocular tubercle absent; cranial crest absent; (5) choanae small, ovoid; partially concealed by palatal shelf of maxillary arch; dentigerous processes of vomers prominent, triangular in outline, separated medially for a distance equal to half the width of the visible dentigerous process, positioned posterior to level of choanae, each dentigerous process of vomers bearing five to seven teeth; (6) males lacking vocal slits, small non-spinous nuptial pads on median surface of thumb above joint with metacarpal; (7) first finger equal or slightly longer than second; circumferential grooves present on all fingers; discs slightly wider than digits, disc on finger I equal to disc on finger II and these in turn smaller than discs on fingers III and IV; morphology of the ungual flap on fingers pointed; (8) fingers with lateral fringes, weakly defined on outer side of finger 4; palmar tubercle bifid (inner lobe largest); thenar tubercle oval, slightly smaller than inner lobe of palmar tubercle; supernumerary tubercles prominent, two on thumb and third finger and one on second and fourth; subarticular tubercles present; subarticular tubercles projecting, with elongate base and larger than supernumerary tubercles; (9) three low ulnar tubercles not coalesced; antebrachial tubercle present; (10) small tubercles on heel and outer edge of tarsus; inner tarsal fold absent; (11) elongate inner metatarsal tubercle, its length twice its width; protuberant, conical outer metatarsal tubercle one-half size of inner metatarsal tubercle; supernumerary plantar tubercles absent; subarticular tubercles present; subarticular tubercles projecting, with elongate base; (12) toes bearing prominent lateral fringes, in finger III and IV only visible in the distal part of digits; toe webbing absent; toe III much longer than toe V; toe III extending to penultimate subarticular tubercle of toe IV; toe V reaching two-thirds between basal and penultimate subarticular tubercles of toe IV; discs and circumferential grooves present on all toes; discs of toes II–IV larger than discs on fingers I and V; (13) color in life: dorsum reddish-brown to grayish, with or without two dark brown marks originating at the snout, extending through the loreal region and upper eyelids, and continuing along the \ /-shaped scapular folds to converge at the sacral region. Supratympanic fold with a dark mark extending to the upper edge of the cream-colored postrictal ridge. A broad dark mark, bordered by a cream coloration, extends along the outer edge of the dorsolateral folds; it becomes wider at the posterior ends, where the supra-inguinal spots are located. Black anal patch present. Diffuse brown labial bars and marked transverse brown bars on the arms and legs. The groin, and anterior and posterior surfaces of the thighs are reddish-orange, the anterior part of flanks is yellow, and the ventral surfaces of thighs, belly, and arms are yellow. The throat is brown with silvery-gray flecks, featuring a superficial yellow coloration restricted to its medial portion that does not extend to the margins. However, the intensity of this yellow and orange coloration may vary among individuals; specifically, it can be much less intense (paler) in juveniles, subadults, and some females. Ventral surfaces of hands and feet, dark. Iris bright copper with black flecks; (14) males smaller than females; SVL in adult males 21.5–25.1 mm (mean ± 1 SD = 23.7 ± 1.3 mm; *n* = 6) and 31.1 mm in one female.

###### Comparisons with congeners.

*Niceforonia tempestuosa* is distinctive in the genus by having dorsolateral folds, tympanic membrane, fleshy swelling of lip in adult males, nuptial pads, circumferential grooves on all fingers, black facemask on loreal region, dorsum reddish-brown to grayish with two dark brown marks, ventral surfaces of thighs, belly, and arms, yellow, black anal patch, and dark supra-inguinal spots. *Niceforonia tempestuosa* most closely resembles *N. mantipus* and *N. nigrovittata* by having dorsolateral folds and \ /-shaped scapular folds, tympanic membrane, snout subacuminate in dorsal view, fleshy swelling of lip in adult males, dark supra-inguinal spots, venter smooth and by lacking discoidal fold and vocal slits. Nevertheless, *Niceforonia tempestuosa* is distinguished by having a yellow belly and ventral surfaces of the arms and thighs (light gray background with silvery flecks in *N. mantipus*), and a reddish-orange coloration on the groin and the anterior and posterior surfaces of the thighs (dark brown with silvery and coppery-red flecks in *N. mantipus*). Furthermore, males of the new species exhibit a brown throat with silvery-gray flecks and a superficial yellow coloration restricted to its medial portion that does not extend to the margins, whereas males of *N. mantipus* have a light gray throat with silvery flecks and two well-defined, large dark blotches on the lateral base of the throat (Figs 4, 5). Additionally, *Niceforonia tempestuosa* is distinguished by having nuptial pads (absent in *N. nigrovittata*) and a reddish-orange posterior surface of the thighs (brown with cream flecks in *N. nigrovittata*) (Lynch and Duellman 1980).

*Niceforonia tempestuosa* can be distinguished from the remaining species of the genus by having circumferential grooves on all fingers (absent in fingers I and II in *N. adenobrachia*Ardila-Robayo, Ruiz-Carranza & Barrera-Rodriguez, 1996, *N. brunnea*, *N. latens* Lynch, 1989, *N. nana*, and *N. peraccai*). Furthermore, *Niceforonia tempestuosa* is distinguished from *N. adenobrachia*, *N. brunnea*, and *N. peraccai* by having a black anal patch, and from *N. latens* and *N. nana* by the presence of a tympanic membrane. The development of dorsolateral folds distinguishes *N. tempestuosa* from *N. aderca* (Lynch, 2003), *N. elassodiscus*, *N. fallaciosa* (Duellman, 2000), and *N. philippi*. Additionally, *N. tempestuosa* lacks vocal slits (present in *N. aderca*, *N. dracula*, *N. philippi*, and *N. quilloturo*) and discoidal fold (present in *N. dracula*, *N. elassodiscus*, *N. fallaciosa*, *N. quilloturo*) (Reyes-Puig et al. 2026). The presence of dark supra-inguinal spots distinguishes *N. tempestuosa* from *N. babax* (Lynch, 1989) and *N. lucida* (Cannatella, 1984); these species further differ from *N. tempestuosa* by having vocal slits. *Niceforonia tempestuosa* differs from *N. columbiana* (Werner, 1899) by lacking basal webbing (Lynch 1975), and from *N. araiodactyla* (Duellman & Pramuk, 1999) and *N. elassodiscus* by lacking a discoidal fold (Duellman and Pramuk 1999). Furthermore, the presence of scattered small warts on a smooth dorsum distinguishes *N. tempestuosa* from *N. araiodactyla*, which has uniformly smooth dorsal skin (Duellman and Pramuk 1999). Additionally, the reddish-orange coloration on the posterior surface of the thighs distinguishes *N. tempestuosa* from *N. elassodiscus* (which has a uniform gray to brown coloration on the posterior surface). Finally, *Niceforonia tempestuosa* is genetically highly divergent from all other species in the genus, with uncorrected *p*-distances in 16S gene ranging from 7.44–18.33% (Table 2).

###### Descriptions of the holotype.

An adult male with head slightly wider than body; HW 40.8% of SVL; HL 35.1% of SVL; snout long, subacuminate in dorsal view, rounded in profile; eye-nostril distance 82.7% of diameter of eye; nostrils protuberant, directed slightly back. Canthus rostralis straight in dorsal view, not elevated; loreal region depressed; top of head flat; upper eyelid bearing minute tubercles, its width 62.1% of IOD; supratympanic fold extending only just posterior to tympanic annulus; tympanic membrane present, upper edge of tympanic annulus covered by supratympanic fold; postrictal tubercles fused into ridge. Choanae small, ovoid; partially concealed by palatal shelf of maxillary arch; dentigerous processes of vomers prominent, each process bearing seven teeth; tongue longer than wide, its posterior border not notched, posterior fourth not adherent to floor of mouth; vocal slits absent.

Skin of dorsum smooth with scattered small warts; dorsolateral and \ /-shaped scapular folds present; skin on belly, and throat smooth (Figs 4A–D, 5A–C); discoidal fold absent, no tubercles in the cloacal region. Three low ulnar tubercles on the ventrolateral edge of the forearm, not coalesced; palmar tubercle bifid (inner lobe largest); thenar tubercle oval, slightly smaller than inner lobe of palmar tubercle; subarticular tubercles projecting, with elongate base, one on thumb and second finger and two on third and fourth fingers, and larger than supernumerary tubercles; supernumerary tubercles prominent, two on thumb and third finger and one on second and fourth. Small nuptial pads on median surface of thumb above joint with metacarpal.

Fingers having lateral fringes, weakly defined on outer side of finger 4; relative lengths of fingers I > II < IV < III, all fingers having terminal ventral pads well defined by circumferential grooves; disc on thumb equal to disc on finger II; discs on fingers III and IV smaller than tympanic membrane; morphology of the ungual flap on fingers pointed. Short robust hind limbs; when hind limbs flexed against axis of body, heels touching or barely overlapping; tibia length 54.7% of SVL; foot length 55.9% of SVL; heel and outer edge of tarsus having small tubercles; inner tarsal fold absent; inner metatarsal tubercle elongate, its length twice its width; conical outer metatarsal tubercle one-half size of inner metatarsal tubercle; toes bearing lateral fringes and discs on expanded pads; webbing absent; relative lengths of toes I < II < V < III < IV, toe III much longer than toe V, toe III extending to penultimate subarticular tubercle of toe IV, toe V reaching two thirds between basal and penultimate subarticular tubercles of toe IV (Figs 5B, 6C), all toes having terminal ventral pads well defined by circumferential grooves; discs of toes II–IV larger than discs on fingers I and V; subarticular tubercles projecting, with elongate base, one on toes I and II, two on toes III and V, and three on toe IV; supernumerary plantar tubercles absent.

In life, dorsum brown with two dark brown marks originating at the snout and extending through the loreal region and upper eyelids, where they become diffuse, and continuing along the \ /-shaped scapular folds (without converging). A weak dark mark, bordered by a cream coloration, extends along the outer edge of the dorsolateral folds; it becomes wider at the posterior ends, where the supra-inguinal spots are located. Postrictal ridge cream-colored; labial bars diffuse and brown; arms and legs with marked transverse brown bars. Black anal patch bordered by a cream coloration. Groin, and anterior and posterior surfaces of thighs, reddish-orange; anterior part of flanks, yellow. Ventral surfaces of thighs, belly, and arms, yellow with uniformly distributed dark brown marks (more abundant on the flanks and anterior part of the belly). The throat is brown with silvery-gray flecks, featuring a superficial yellow coloration restricted to its medial portion that does not extend to the margins (Fig. 4C, D). Iris bright copper with black flecks (Fig. 4A, B).

###### Measurements of holotype (mm).

SVL 24.5, tibia length 13.4, foot length 13.7, HL 8.7, HW 9.9, IOD 2.9, tympanum diameter 1.3, internarial distance 3.0, width of upper eyelid 1.8, diameter of eye 2.9, eye-nostril distance 2.4.

###### Variation.

Sexual dimorphism in size, with adult females being larger than adult males (Table 3). Dorsal coloration also varies, ranging from reddish-brown to grayish-brown. The same applies to the longitudinal dark markings extending from the snout to the sacral region, which are present in some specimens. We also noted variation in the yellow and orange coloration of ventral surfaces can be much less intense (paler) in juveniles, subadults, and some females. In contrast, the dark supra-inguinal spots are consistently present in all specimens. The fleshy swelling of the lip is only evident in adult males.

**Table 3.** Selected meristic, mensural (in mm), and proportional (in percentages) data from the type series of *Niceforonia tempestuosa*.

**Character**	**Male (*n* = 6)**	**Female (*n* = 1)**
Snout-vent length	21.5–25.1 (23.7 ± 1.3)	31.1
Head width	8.6–10.0 (9.5 ± 0.5)	11.8
Head length	7.8–8.7 (8.4 ± 0.3)	10.8
Interorbital distance	2.1–2.9 (2.4 ± 0.3)	2.7
Upper eyelid width	1.5–1.8 (1.7 ± 0.1)	2.3
Eye diameter	2.4–2.9 (2.6 ± 0.1)	3.4
Eye-nostril distance	2.0–2.4 (2.1 ± 0.1)	2.3
Internarial distance	2.6–3.0 (2.8 ± 0.2)	3.5
Tympanum diameter	0.9–1.3 (1.0 ± 0.1)	1.6
Tibia length	11.9–14.1 (12.9 ± 0.7)	16.2
Foot length	12.0–13.7 (13.1 ± 0.6)	16.8
Head length / Snout–vent length	34.6–35.7	34.7
Head width / Snout–vent length	39.0–40.8	37.9
Eye diameter / Head length	30.7–33.3	31.4
Eye-nostril distance / Eye diameter	74.0–87.5	67.6
Upper eyelid width / Interorbital distance	62.0–80.9	85.1
Interorbital distance / Head width	22.8–29.2	22.8
Tympanum diameter / Eye diameter	33.3–44.8	47.0
Tibia length / Snout–vent length	52.5–56.1	52.0
Foot length / Snout–vent length	54.1–55.9	54.0

###### Distribution and ecology.

**Figure 7.**
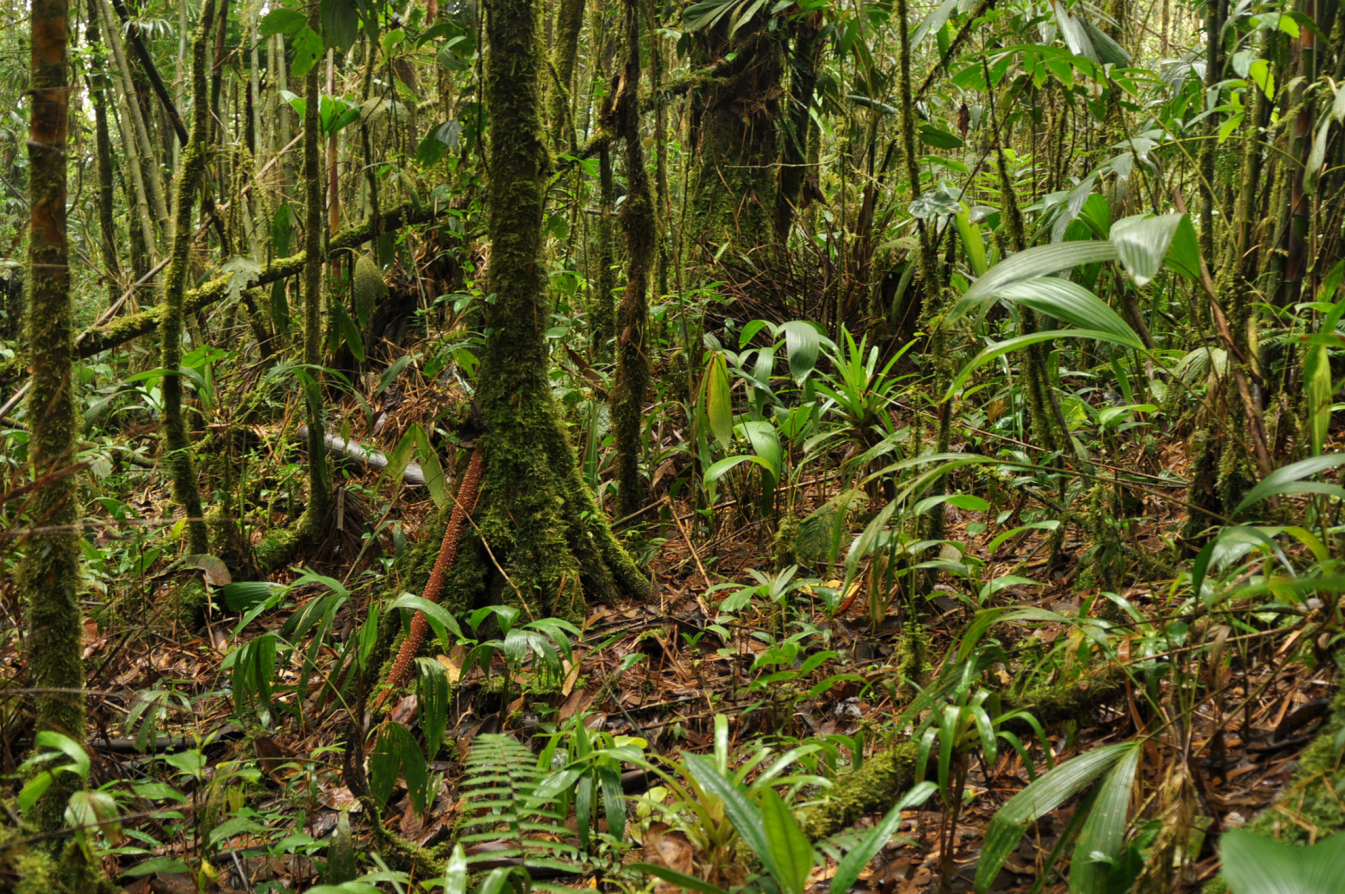
Habitat of *Niceforonia tempestuosa* at the type locality. The habitat is characterized by the presence of large tree buttresses and stilt roots of *Wettinia* sp. palms, with the forest floor covered by abundant leaf litter.

The species is known only from two localities in humid montane forests of the central part of the Cordillera Occidental of Colombia, within the Key Biodiversity Area (KBA) Serranía de los Paraguas: Reserva Natural Comunitaria Cerro El Inglés (El Cairo, Valle del Cauca) and El Mirador (Balsal, Versalles, Valle del Cauca, 4°40'N, 76°17'W), between 2169–2294 m (Fig. 1). The species inhabits the leaf litter of well-preserved forest interiors, where it can be found by disturbing the substrate during the day or actively foraging at night. The species exhibits a preference for sloping over level microhabitats. Individuals have also been observed within crevices formed by the buttress roots of large trees and among the stilt roots of *Wettinia* sp. palms (Fig. 7). As a leaf-litter dweller, this species is syntopic with *Strabomantis cerastes*, another inhabitant of the forest floor in the Serranía de los Paraguas.

###### Etymology.

The specific epithet is the feminine adjective tempestuosa (meaning “stormy”), derived from the Latin noun *tempestas* (meaning “storm” or “weather”). This name was chosen for local communities as a metaphor for the global climate emergency, referring to the increased frequency and intensity of extreme weather events—symbolized by the “storm”—which represent a growing threat to global biodiversity. The discovery of this new species in the Key Biodiversity Area (KBA) Serranía de los Paraguas underscores the resilience of this biodiversity hotspot amidst the current climate crisis, safeguarded by the persistent efforts of local communities and biodiversity guardians.

###### Remarks.

Lynch and Duellman (1980), Lynch (1989), and Lynch (2003) reported the occurrence of a fleshy swelling of the upper lip in adult males of *N. nigrovittata*, *N. mantipus*, and *N. aderca*, respectively, followed more recently by Reyes-Puig et al. (2026) for *N. quilloturo*. We herein report this character in males of *Niceforonia latens* and *Niceforonia tempestuosa*. According to Lynch (2003), the presence of this character exclusively in males suggests an involvement in either courtship or the excavation of egg burrows. Among brachycephaloid frogs, a character resembling the fleshy swelling of the upper lip found in *Niceforonia* occurs in species of the genus *Eleutherodactylus*, subgenus *Pelorius* (Hedges et al. 2008a). As noted by Hedges et al. (2008a), species possessing shovel-shaped snouts are specialized in constructing enclosed underground chambers for vocalization and, presumably, oviposition. To date, the courtship and nesting behaviors of *Niceforonia* species remain unknown; therefore, the functional implications of this labial swelling warrant further investigation.

### Conservation and threats

The Key Biodiversity Area (KBA) Serranía de los Paraguas hosts more than 100 amphibian species, 32 of which are threatened with extinction according to the IUCN Red List. Although amphibians in this area have been studied since the 1980s, new localities within this biodiversity hotspot have only recently begun to be explored. Given this, both a greater amphibian diversity and a broader distribution for *Niceforonia tempestuosa* could exist within the region. This area is currently impacted by habitat loss driven by illicit crop expansion, logging, agricultural frontier expansion (e.g., Hass avocado farming), and increasing pressure from large-scale mining interests. These extractive projects are deceptively promoted under the narrative of local development, masking their severe ecological toll and long-term socio-environmental damage. These threats have escalated in recent years, largely driven by institutional absence and persistent socio-political challenges in Colombia. To confront all these challenges, a joint conservation initiative led by Fundación Calima and the local community organization Corporación Serraniagua has been ongoing since 2019, supported by The Rufford Foundation, the Prince Bernhard Nature Fund (PBNF), and the Critical Ecosystem Partnership Fund (CEPF). This initiative combines acoustic, climatic, and field monitoring, basic research, the identification and protection of breeding habitats, and community outreach activities. These efforts specifically aim to empower local populations with the knowledge needed to make informed decisions regarding development projects proposed within their territories. Among the actions performed to protect habitats and increase the resilience of amphibian diversity in this hotspot, zoning strategies have been implemented to regulate the impact of scientific research and ecotourism. Specifically for *Niceforonia tempestuosa*, these measures focus on restricting foot traffic in areas with dense leaf litter, where the species reaches its highest population densities. Furthermore, in coordination with local landowners, fences are being installed to safeguard riparian forests and streams from the encroachment of the agricultural and livestock frontier, complemented by signage that informs visitors of mitigation protocols in these sensitive areas.

### Evaluation of diagnostic character states

In the context of the phylogenetic hypothesis favored herein (Fig. 3), we found that the occurrence of dark supra-inguinal spots is optimized as a synapomorphy of a clade within the genus *Niceforonia* that includes *N. brunnea*, *N. dracula*, *N. elassodiscus*, *N. mantipus*, *N. nigrovittata*, *N. quilloturo*, *N. peraccai*, four candidate species, and *N. tempestuosa*. The occurrence of dark supra-inguinal spots also optimizes as a synapomorphy of a well-supported clade (Jackknife = 99%) within Holoadeninae composed of the genera *Bahius*, *Barycholos*, *Phyllonastes*, and *Urkuphryne* (Fig. 3). These optimizations are also supported by the ML topology (Suppl. material 4).

## Discussion and conclusions

The phylogenetic position of *Niceforonia* within the superfamily Brachycephaloidea has been shown to be unstable. For example, it was recognized within the subfamily Strabomantinae by Hedges et al. (2008a), albeit with low nodal support. Subsequently, Pyron and Wiens (2011) recovered it as sister to a clade comprising the genus *Strabomantis* (Strabomantinae) and the family Craugastoridae, although this relationship was weakly supported. Padial et al. (2014) later assigned it to the subfamily Holoadeninae, a placement that received high nodal support. Furthermore, it was recovered as a member of the newly recognized subfamily Hypodactylinae, which was placed as sister to a clade composed of the subfamilies Strabomantinae, Holoadeninae, and Pristimantinae by Heinicke et al. (2018), a placement that received strong nodal support. More recently, Portik et al. (2023) recovered it as a sister clade to Strabomantinae, a relationship that received moderate support. Within this context, our phylogeny shows that *Niceforonia* is not part of the subfamilies Holoadeninae or Strabomantinae. This is consistent with the findings of Pyron and Wiens (2011), Heinicke et al. (2018), and Portik et al. (2023), although the relationships among these subfamilies remain contentious. Analyzed together, this evidence clearly indicates a genuine evolutionary or methodological conflict rather than a mere lack of phylogenetic resolution, an issue that must be tested more rigorously. This will involve not only increased taxon sampling but also the essential incorporation of phenotypic data into total evidence analyses. Such an integration is critical, as combining phenotypic and molecular evidence is a useful approach for dealing with phylogenetic incongruence (e.g., Koch and Thompson 2020; Neumann et al. 2020), a necessity highlighted by the conflicting phylogenetic signals regarding the genus *Niceforonia*.

While resolving the deep-level placement of the genus remains a macro-systematic challenge, fine-scale evolutionary relationships within *Niceforonia* also exhibit subtle variations across studies. When comparing our in-group topology with the most recent contribution by Reyes-Puig et al. (2026), we find a high degree of overall topological congruence, with differences restricted to only two specific clades (Fig. 3). First, regarding the relationships of *N. peraccai*, Reyes-Puig et al. (2026) recovered this taxon as sister to *N.* sp. 2. In contrast, our phylogeny places *N. peraccai* as sister to a broader clade composed of *N.* sp. 2, *N.* sp. 3, *N.* sp. 4, and *N. quilloturo*. Second, we observe a discrepancy in the relationships among *N. brunnea*, *N. dracula*, and *N. elassodiscus*. Reyes-Puig et al. (2026) recovered *N. elassodiscus* as sister to *N. dracula*, which together form the sister group to a clade comprising *N. brunnea*, *N. peraccai*, *N.* sp. 2, *N.* sp. 3, *N.* sp. 4, and *N. quilloturo*. Conversely, in our phylogeny, *N. elassodiscus* is sister to a clade composed of *N. brunnea* and *N. dracula*, which in turn is sister to the clade containing *N. peraccai*, *N.* sp. 2, *N.* sp. 3, *N.* sp. 4, and *N. quilloturo*.

These topological and phylogenetic discrepancies, which may be attributed to differences in taxon and character sampling, as well as the distinct inference methods employed, further highlight the necessity of pursuing a comprehensive taxonomic revision of the genus *Niceforonia*. As demonstrated by the recent findings of Reyes-Puig et al. (2026) in Ecuador, it is highly likely that undescribed, cryptic species remain hidden under the names of already recognized taxa. In the case of Colombia, the species we describe herein was long recognized as *Niceforonia mantipus*, a widely distributed Colombian species. To refine the delimitation of *N. mantipus*, we incorporated, for the first time, samples from the Valle del Cauca (Western Cordillera) and Quindío (Central Cordillera) departments in Colombia, including material from the type locality. These samples were recovered as closely related (Fig. 3). Given that *N. mantipus* also occurs in the south (Cauca) and north (Antioquia), further assessment of the taxonomic status of these populations is necessary. In this context, integrating phenotypic and molecular evidence is essential for a taxonomic revision of *Niceforonia*, as it allows for the delimitation of cryptic species and ensures that the nomenclature accurately reflects both its diversity and evolutionary history.

### Phylogenetic significance of dark supra-inguinal spots in Strabomantidae

Our results demonstrate that the occurrence of dark supra-inguinal spots is phylogenetically informative, diagnosing two clades within the family Strabomantidae. The first clade appears within the genus *Niceforonia* and includes *N. brunnea*, *N. dracula*, *N. elassodiscus*, *N. mantipus*, *N. nigrovittata*, *N. quilloturo*, *N. peraccai*, four candidate species, and *N. tempestuosa* (Fig. 3). This character optimization is consistent with the topology proposed by Portik et al. (2023), where *N. brunnea*, *N. peraccai*, and *N. nigrovittata* form a clade that does not include *N. nana* and *N. philippi*, and the genus *Niceforonia* is sister to *Strabomantis*. Following the proposal of Lynch (1989), we maintain the name *Niceforonia nigrovittata* species group to recognize this clade, diagnosed by the occurrence of dark supra-inguinal spots, thus maintaining stability and the well-established historical use of this name in the literature. We also assessed the distribution of dark supra-inguinal spots among *Niceforonia* species for which molecular data are not available, finding that this character appears in *N. aderca* and *N. fallaciosa*. Consequently, we propose including these two species in the *N. nigrovittata* species group. The inclusion of these species in the group is tentative and requires rigorous testing using molecular data, as current evidence suggests that supra-inguinal spots also occur in genera other than *Niceforonia*, such as *Barycholos*, *Noblella*, *Phrynopus*, and *Phyllonastes* within the family Strabomantidae.

The second clade where the occurrence of dark supra-inguinal spots optimizes as a synapomorphy appears within the subfamily Holoadeninae and includes the genera *Bahius*, *Barycholos*, *Phyllonastes*, and *Urkuphryne* (Fig. 3). This character optimization is consistent with the recent work of Ortega et al. (2025), whose topology at the level of relationships among genera for Holoadeninae was also recovered in our analysis. Regarding the clade composed of these four genera, Ospina-Sarria and Grant (2022) previously proposed a synapomorphy for a clade composed of *Phyllonastes* + *Barycholos* based on the posterior origin of the *m. iliacus externus* on the iliac shaft. Considering this evidence, it is necessary to assess the origin of the *m. iliacus externus* in the recently described genus *Urkuphryne*, which will clarify the distribution of this nested phenotypic synapomorphy within the subfamily Holoadeninae. Analyzed together, anatomical (origin of the *m. iliacus externus*), molecular (DNA sequences), and morphological (dark supra-inguinal spots) evidence corroborates the monophyly of this group within the subfamily Holoadeninae, granting multi-level resolution and precise taxonomic diagnoses that demonstrate the integration of molecular and phenotypic evidence as a powerful tool for testing phylogenetic hypotheses in brachycephaloid frogs.

## Supplementary Material

XML Treatment for Niceforonia tempestuosa

## Additional information

### Artificial Intelligence (AI) use

The authors accept full responsibility for the content of the manuscript, including the disclosure of any use of AI.

No AI tools were used in the preparation of this manuscript.

### Author ORCIDs

J.J. Ospina-Sarria https://orcid.org/0000-0002-9099-7793

A.F. Rodríguez-Betancourt https://orcid.org/0000-0002-0298-0677

X. Gómez-Sepúlveda https://orcid.org/0000-0001-5582-0709

I.D. Morales-Vertel https://orcid.org/0009-0008-4465-4432

V. Vélez-Franco https://orcid.org/0009-0003-3148-2831

N. Gallego-Posada https://orcid.org/0009-0000-5113-7531

J.D. Herrera-Rengifo https://orcid.org/0009-0001-5683-6558

J.E. Grajales Escobar https://orcid.org/0009-0006-0271-7285

I. Ceballos-Castro https://orcid.org/0000-0001-7478-7836

I. García-Gómez https://orcid.org/0000-0003-3326-0290

D.A. Velásquez-Trujillo https://orcid.org/0000-0001-9971-8431

A.S. Ortiz Baez https://orcid.org/0000-0003-0080-0633

J. Mendez Narvaez https://orcid.org/0000-0001-6127-3377

### Data availability

All of the data that support the findings of this study are available in the main text or Supplementary Information.

## Supplementary materials

Supplementary material 1 List of examined specimens Data type docx Explanation note List of examined specimens (specimens with institutional collection numbers were physically examined by the lead author), taxonomic distribution of dark supra-inguinal spots, and data sources. https://binary.pensoft.net/file/1742765This dataset is made available under the Open Database License (http://opendatacommons.org/licenses/odbl/1.0/). The Open Database License (ODbL) is a license agreement intended to allow users to freely share, modify, and use this Dataset while maintaining this same freedom for others, provided that the original source and author(s) are credited. Jhon Jairo Ospina-Sarria, Andrés Felipe Rodríguez-Betancourt, Ximena Gómez-Sepúlveda, Iván Darío Morales-Vertel, Valentina Vélez-Franco, Nayely Gallego-Posada, Jose Daniel Herrera-Rengifo, John Edison Grajales Escobar, Irene Ceballos-Castro, Isabella García-Gómez, David Andrés Velásquez-Trujillo, Ayda Susana Ortiz Baez, Javier Mendez Narvaez

Supplementary material 2 GenBank accession numbers, voucher specimens, and molecular markers Data type docx Explanation note GenBank accession numbers, voucher specimens, and molecular markers used in this study. New sequences generated for this study are indicated in bold. https://binary.pensoft.net/file/1742766This dataset is made available under the Open Database License (http://opendatacommons.org/licenses/odbl/1.0/). The Open Database License (ODbL) is a license agreement intended to allow users to freely share, modify, and use this Dataset while maintaining this same freedom for others, provided that the original source and author(s) are credited. Jhon Jairo Ospina-Sarria, Andrés Felipe Rodríguez-Betancourt, Ximena Gómez-Sepúlveda, Iván Darío Morales-Vertel, Valentina Vélez-Franco, Nayely Gallego-Posada, Jose Daniel Herrera-Rengifo, John Edison Grajales Escobar, Irene Ceballos-Castro, Isabella García-Gómez, David Andrés Velásquez-Trujillo, Ayda Susana Ortiz Baez, Javier Mendez Narvaez

Supplementary material 3 Phylogenetic relationships of *Niceforonia* and outgroups Data type pdf Explanation note Phylogenetic relationships of *Niceforonia* and outgroups recovered in one of the 97 most-parsimonious trees of 24,174 steps from a parsimony analysis in TNT treating gaps as a fifth state. Numbers on nodes are Parsimony jackknife absolute frequencies values. Nodes without values indicate value < 70% of jackknife value. Character states with primitive-derived states are indicated within the squares (see text and Suppl. material 1 for character definitions). Color-coding: red and blue highlight the genus *Niceforonia* and a clade comprising of *Bahius*, *Barycholos*, *Phyllonastes*, and *Urkuphryne*, respectively; red squares indicate unique, homoplastic. https://binary.pensoft.net/file/1742767This dataset is made available under the Open Database License (http://opendatacommons.org/licenses/odbl/1.0/). The Open Database License (ODbL) is a license agreement intended to allow users to freely share, modify, and use this Dataset while maintaining this same freedom for others, provided that the original source and author(s) are credited. Jhon Jairo Ospina-Sarria, Andrés Felipe Rodríguez-Betancourt, Ximena Gómez-Sepúlveda, Iván Darío Morales-Vertel, Valentina Vélez-Franco, Nayely Gallego-Posada, Jose Daniel Herrera-Rengifo, John Edison Grajales Escobar, Irene Ceballos-Castro, Isabella García-Gómez, David Andrés Velásquez-Trujillo, Ayda Susana Ortiz Baez, Javier Mendez Narvaez

Supplementary material 4 Phylogenetic relationships of *Niceforonia* and outgroups recovered in the maximum likelihood analysis with IQ-TREE Data type pdf Explanation note Phylogenetic relationships of *Niceforonia* and outgroups recovered in the maximum likelihood analysis with IQ-TREE (log-likelihood of the best ML tree: -105312.6350). Numbers around nodes are the ultrafast bootstrap support values. Color-coding: red and blue highlight the genus *Niceforonia* and a clade comprising of *Bahius*, *Barycholos*, *Phyllonastes* and *Urkuphryne*, respectively. https://binary.pensoft.net/file/1742768This dataset is made available under the Open Database License (http://opendatacommons.org/licenses/odbl/1.0/). The Open Database License (ODbL) is a license agreement intended to allow users to freely share, modify, and use this Dataset while maintaining this same freedom for others, provided that the original source and author(s) are credited. Jhon Jairo Ospina-Sarria, Andrés Felipe Rodríguez-Betancourt, Ximena Gómez-Sepúlveda, Iván Darío Morales-Vertel, Valentina Vélez-Franco, Nayely Gallego-Posada, Jose Daniel Herrera-Rengifo, John Edison Grajales Escobar, Irene Ceballos-Castro, Isabella García-Gómez, David Andrés Velásquez-Trujillo, Ayda Susana Ortiz Baez, Javier Mendez Narvaez
