# Supplementary material for: A new species of Niceforonia (Amphibia, Anura, Strabomantidae) from the Serranía de los Paraguas, Colombia, with the description of a new phenotypic synapomorphy for the Niceforonia nigrovittata species group
Source: Zookeys. 2026 Aug 11;1289:159–84. doi: 10.3897/zookeys.1289.198411 (PMC13487511; doi:10.3897/zookeys.1289.198411)
Supplement: Supplementary file 2 [file zookeys-1289-159_article-198411__-s004.pdf]

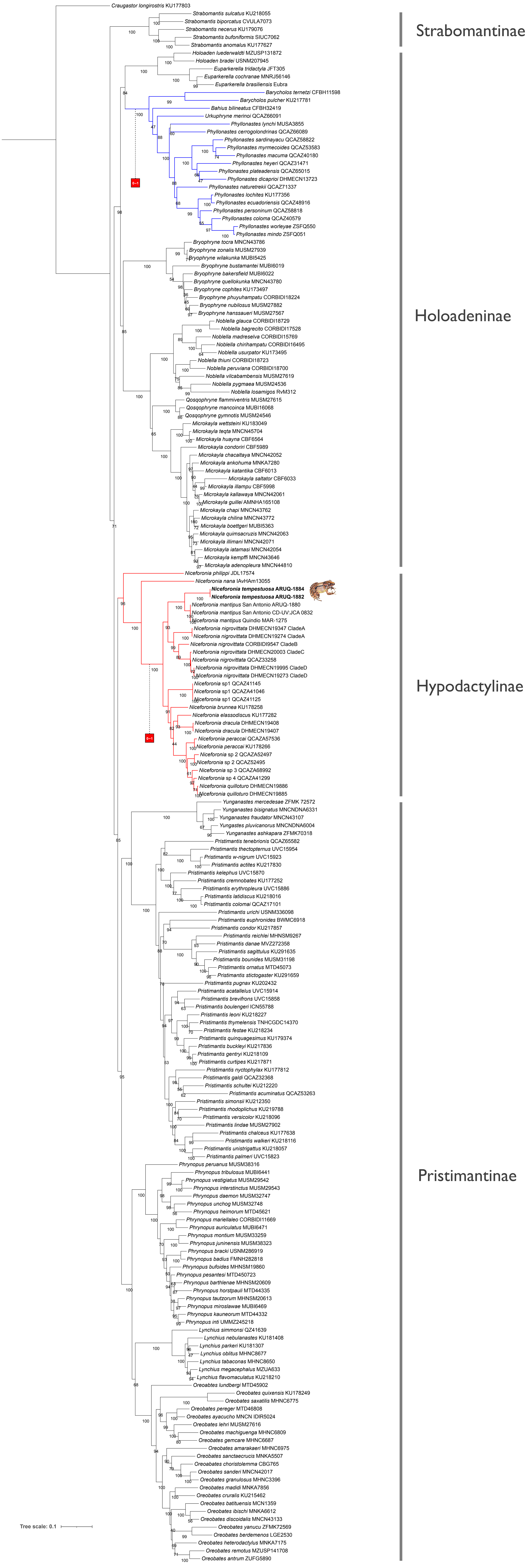

Craugastor longirostris KU177803
Strabomantis sulcatus KU218055
Strabomantis biporcatus CVULA7073
Strabomantis necerus KU179076
Strabomantis bufoniformis SIUC7062
Strabomantis anomalus KU177627
Holoaden luederwaldti MZUSP131872
Holoaden bradei USNM207945
Euparkerella tridactyla JFT305
Euparkerella cochranae MNRJ56146
Euparkerella brasiliensis Eubra
Barycholos ternetzi CFBH11598
Barycholos pulcher KU217781
Bahius bilineatus CFBH32419
Urkuphryne merinoi QCAZ66091
Phyllonastes lynchi MUSA3855
Phyllonastes cerrogolondrinas QCAZ66089
Phyllonastes sardinayacu QCAZ58822
Phyllonastes myrmecoides QCAZ53583
Phyllonastes macuma QCAZ40180
Phyllonastes heyeri QCAZ31471
Phyllonastes plateadensis QCAZ65015
Phyllonastes dicaprioi DHMECN13723
Phyllonastes naturetrekii QCAZ71337
Phyllonastes lochites KU177356
Phyllonastes ecuadoriensis QCAZ48916
Phyllonastes personinum QCAZ58818
Phyllonastes coloma QCAZ40579
Phyllonastes worleyae ZSFQ550
Phyllonastes mindo ZSFQ051
Bryophryne tocra MNCN43786
Bryophryne zonalis MUSM27939
Bryophryne wilakunka MUBI5425
Bryophryne bustamantei MUBI6019
Bryophryne bakersfield MUBI6022
Bryophryne quellokunka MNCN43780
Bryophryne cophites KU173497
Bryophryne phuyuhampatu CORBIDI18224
Bryophryne nubilosus MUSM27882
Bryophryne hanssaueri MUSM27567
Noblella glauca CORBIDI18729
Noblella bagrecito CORBIDI17528
Noblella madreselva CORBIDI15769
Noblella chirihampatu CORBIDI16495
Noblella usurpator KU173495
Noblella thiuni CORBIDI18723
Noblella peruviana CORBIDI18700
Noblella vilcabambensis MUSM27619
Noblella pygmaea MUSM24536
Noblella losamigos RvM312
Qosqophryne flammiventris MUSM27615
Qosqophryne mancoinca MUBI16068
Qosqophryne gymnotis MUSM24546
Microkayla wettsteini KU183049
Microkayla teqta MNCN45704
Microkayla huayna CBF6564
Microkayla condoriri CBF5989
Microkayla chacaltaya MNCN42052
Microkayla ankohuma MNKA7280
Microkayla katantika CBF6013
Microkayla saltator CBF6033
Microkayla illampu CBF5998
Microkayla kallawaya MNCN42061
Microkayla guillei AMNHA165108
Microkayla chapi MNCN43762
Microkayla chilina MNCN43772
Microkayla boettgeri MUBI5363
Microkayla quimsacruzis MNCN42063
Microkayla illimani MNCN42071
Microkayla iatamasi MNCN42054
Microkayla kempffi MNCN43646
Microkayla adenopleura MNCN44810
Niceforonia philippi JDL17574
Niceforonia nana IAvHAm13055
Niceforonia tempestuosa ARUQ-1884
Niceforonia tempestuosa ARUQ-1882
Niceforonia mantipus San Antonio ARUQ-1880
Niceforonia mantipus San Antonio CD-UV:JCA 0832
Niceforonia mantipus Quindio MAR-1275
Niceforonia nigrovittata DHMECN19347 CladeA
Niceforonia nigrovittata DHMECN19274 CladeA
Niceforonia nigrovittata CORBIDI9547 CladeB
Niceforonia nigrovittata DHMECN20003 CladeC
Niceforonia nigrovittata QCAZ33258
Niceforonia nigrovittata DHMECN19995 CladeD
Niceforonia nigrovittata DHMECN19273 CladeD
Niceforonia sp1 QCAZ41145
Niceforonia sp1 QCAZA41046
Niceforonia sp1 QCAZ41125
Niceforonia brunnea KU178258
Niceforonia elassodiscus KU177282
Niceforonia dracula DHMECN19408
Niceforonia dracula DHMECN19407
Niceforonia peraccai QCAZA57536
Niceforonia peraccai KU178266
Niceforonia sp 2 QCAZA52497
Niceforonia sp 2 QCAZ52495
Niceforonia sp 3 QCAZA68992
Niceforonia sp 4 QCAZA41299
Niceforonia quilloturo DHMECN19886
Niceforonia quilloturo DHMECN19885
Yunganastes mercedesae ZFMK 72572
Yunganastes bisignatus MNCNDNA6331
Yunganastes fraudator MNCN43107
Yungastes pluvicanorus MNCNDNA6004
Yunganastes ashkapara ZFMK70318
Pristimantis tenebrionis QCAZ65582
Pristimantis thectopternus UVC15954
Pristimantis w-nigrum UVC15923
Pristimantis actites KU217830
Pristimantis kelephus UVC15870
Pristimantis cremnobates KU177252
Pristimantis erythropleura UVC15886
Pristimantis latidiscus KU218016
Pristimantis colomai QCAZ17101
Pristimantis urichi USNM336098
Pristimantis euphronides BWMC6918
Pristimantis condor KU217857
Pristimantis reichlei MHNSM9267
Pristimantis danae MVZ272358
Pristimantis sagittulus KU291635
Pristimantis bounides MUSM31198
Pristimantis ornatus MTD45073
Pristimantis stictogaster KU291659
Pristimantis pugnax KU202432
Pristimantis acatallelus UVC15914
Pristimantis brevifrons UVC15858
Pristimantis boulengeri ICN55788
Pristimantis leoni KU218227
Pristimantis thymelensis TNHCGDC14370
Pristimantis festae KU218234
Pristimantis quinquagesimus KU179374
Pristimantis buckleyi KU217836
Pristimantis gentryi KU218109
Pristimantis curtipes KU217871
Pristimantis nyctophylax KU177812
Pristimantis galdi QCAZ32368
Pristimantis schultei KU212220
Pristimantis acuminatus QCAZ53263
Pristimantis simonsii KU212350
Pristimantis rhodoplichus KU219788
Pristimantis versicolor KU218096
Pristimantis lindae MUSM27902
Pristimantis chalceus KU177638
Pristimantis walkeri KU218116
Pristimantis unistrigattus KU218057
Pristimantis palmeri UVC15823
Phrynopus peruanus MUSM38316
Phrynopus tribulosus MUBI6441
Phrynopus vestigiatus MUSM29542
Phrynopus interstinctus MUSM29543
Phrynopus daemon MUSM32747
Phrynopus unchog MUSM32748
Phrynopus heimorum MTD45621
Phrynopus mariellaleo CORBIDI11669
Phrynopus auriculatus MUBI6471
Phrynopus montium MUSM33259
Phrynopus juninensis MUSM38323
Phrynopus bracki USNM286919
Phrynopus badius FMNH282818
Phrynopus bufoides MHNSM19860
Phrynopus pesantesi MTD450723
Phrynopus barthlenae MHNSM20609
Phrynopus horstpauli MTD44335
Phrynopus tautzorum MHNSM20613
Phrynopus miroslawae MUBI6469
Phrynopus kauneorum MTD44332
Phrynopus inti UMMZ245218
Lynchius simmonsi QZ41639
Lynchius nebulanastes KU181408
Lynchius parkeri KU181307
Lynchius oblitus MHNC8677
Lynchius tabaconas MHNC8650
Lynchius megacephalus MZUA633
Lynchius flavomaculatus KU218210
Oreobates lundbergi MTD45902
Oreobates quixensis KU178249
Oreobates saxatilis MHNC6775
Oreobates pereger MTD46808
Oreobates ayacucho MNCN IDIR5024
Oreobates lehri MUSM27616
Oreobates machiguenga MHNC6809
Oreobates gemcare MHNC6687
Oreobates amarakaeri MHNC6975
Oreobates sanctaecrucis MNKA5507
Oreobates choristolemma CBG765
Oreobates sanderi MNCN42017
Oreobates granulosus MHNC3396
Oreobates madidi MNKA7856
Oreobates cruralis KU215462
Oreobates batituensis MCN1359
Oreobates ibischi MNKA6612
Oreobates discoidalis MNCN43133
Oreobates yanucu ZFMK72569
Oreobates berdemenos LGE2530
Oreobates heterodactylus MNKA7175
Oreobates remotus MZUSP141708
Oreobates antrum ZUFG5890
Strabomantinae
Holoadeninae
Hypodactylinae
Pristimantinae
Tree scale: 0.1
