# Supplementary material for: A new species of Niceforonia (Amphibia, Anura, Strabomantidae) from the Serranía de los Paraguas, Colombia, with the description of a new phenotypic synapomorphy for the Niceforonia nigrovittata species group
Source: Zookeys. 2026 Aug 11;1289:159–84. doi: 10.3897/zookeys.1289.198411 (PMC13487511; doi:10.3897/zookeys.1289.198411)
Supplement: Supplementary file 3 [file zookeys-1289-159_article-198411__-s001.docx]

| **Species** | **Occurrence of dark suprainguinal spots: state 0, absent; state 1, present.** | **Source** |
| --- | --- | --- |
|  |  |  |
| *Bahius bilineatus* | 1 | Dias et al. 2017 |
| *Barycholos pulcher* | 1 | KU 146044, 217781, Coloma and Duellman 2025 |
| *Barycholos ternetzi* | ? |  |
| *Bryophryne bakersfield* | 0 | Chaparro Auza et al. 2015 |
| *Bryophryne bustamantei* | 0 | Duellman and Lehr, 2009 |
| *Bryophryne cophites* | 0 | KU 138884, 138885, 138913 |
| *Bryophryne hanssaueri* | 0 | De la Riva et al. 2017 "2018" |
| *Bryophryne nubilosus* | 0 | De la Riva et al. 2017 "2018" |
| *Bryophryne phuyuhampatu* | 0 | Catenazzi et al. 2017 |
| *Bryophryne quellokunka* | 0 | De la Riva et al. 2017 "2018" |
| *Bryophryne tocra* | 0 | De la Riva et al. 2017 "2018" |
| *Bryophryne wilakunka* | 0 | Lehr and Catenazzi 2008 |
| *Bryophryne zonalis* | 0 | Lehr and Catenazzi 2009a |
| *Craugastor longirostris* | 0 | KU 165500, 165502 |
| *Euparkerella brasiliensis* | 0 | Fusinatto et al. 2013 |
| *Euparkerella cochranae* | 0 | Vrcibradic et al. 2008 |
| *Euparkerella tridactyla* | 0 | Ferreira et al. 2010 |
| *Holoaden luederwaldti* | 0 | KU 92869 |
| *Holoaden bradei* | 0 | KU 92865 |
| *Lynchius flavomaculatus* | 0 | KU 165968, 119719 |
| *Lynchius megacephalus* | 0 | Sánchez-Nivicela et al. 2019 |
| *Lynchius nebulanastes* | 0 | KU 181407, 181400, 181408 |
| *Lynchius oblitus* | 0 | Motta et al. 2016 |
| *Lynchius parkeri* | 0 | KU 135278, 135290, 181307 |
| *Lynchius simmonsi* | 0 | KU 147068, 147069, Lynch 1974 |
| *Lynchius tabaconas* | 0 | Motta et al. 2016 |
| *Microkayla adenopleura* | 0 | Aguayo-Vedia and Harvey 2001 |
| *Microkayla ankohuma* | 0 | De la Riva 2007 |
| *Microkayla boettgeri* | 0 | Lehr 2006 |
| *Microkayla chacaltaya* | 0 | De la Riva 2007 |
| *Microkayla chapi* | 0 | De la Riva et al. 2017 "2018" |
| *Microkayla chilina* | 0 | De la Riva et al. 2017 "2018" |
| *Microkayla condoriri* | 0 | De la Riva 2007 |
| *Microkayla guillei* | 0 | De la Riva 2007 |
| *Microkayla huayna* | 0 | De la Riva et al. 2017 |
| *Microkayla iatamasi* | 0 | Aguayo-Vedia and Harvey 2001 |
| *Microkayla illampu* | 0 | De la Riva 2007 |
| *Microkayla illimani* | 0 | De la Riva 2007 |
| *Microkayla kallawaya* | 0 | De la Riva 2007 |
| *Microkayla katantika* | 0 | De la Riva 2007 |
| *Microkayla kempffi* | 0 | KU 220067, 220068 |
| *Microkayla quimsacruzis* | 0 | De la Riva 2007 |
| *Microkayla saltator* | 0 | De la Riva 2007 |
| *Microkayla teqta* | 0 | De la Riva and Burrowes, 2014 |
| *Microkayla wettsteini* | 0 | KU 154558, 183049 |
| *Niceforonia aderca* | 1 | MHN-UIS (not catalogued) |
| *Niceforonia babax* | 0 | KU 200202 |
| *Niceforonia brunnea* | 1 | KU 178259, 178261; Coloma and Duellman 2025 |
| *Niceforonia dracula* | 1 | Reyes-Puig et al. 2026 |
| *Niceforonia elassodiscus* | 0&1 | KU 143415, 130272, 177283, KU 168012, ICN 49666 |
| *Niceforonia fallaciosa* | 1 | KU 212219 |
| *Niceforonia latens* | 0 | ICN 397761 |
| *Niceforonia lucida* | 0 | KU 162435 |
| *Niceforonia mantipus* | 1 | KU 143970, 143970 |
| *Niceforonia nana* | 0 | KU 169123, 169125 |
| *Niceforonia nigrovittata* | 1 | KU 220447, 123527, 123546, 123547 |
| *Niceforonia nigrovittata* – Clade A | ? | Reyes-Puig et al. 2026 |
| *Niceforonia nigrovittata* – Clade B | ? | Reyes-Puig et al. 2026 |
| *Niceforonia nigrovittata* – Clade C | ? | Reyes-Puig et al. 2026 |
| *Niceforonia nigrovittata* – Clade D | ? | Reyes-Puig et al. 2026 |
| *Niceforonia quilloturo* | 1 | Reyes-Puig et al. 2026 |
| *Niceforonia peraccai* | 1 | KU 111430, 178266, 178267 |
| *Niceforonia philippi* | 0 | KU 143505, 165866, 165868 |
| *Niceforonia* sp 1 | ? | Reyes-Puig et al. 2026 |
| *Niceforonia* sp 2 | ? | Reyes-Puig et al. 2026 |
| *Niceforonia* sp 3 | ? | Reyes-Puig et al. 2026 |
| *Niceforonia* sp 4 | ? | Reyes-Puig et al. 2026 |
| *Niceforonia tempestuosa* | 1 | ARUQ-1882–1893 |
| *Noblella bagrecito* | 1 | KU 196514, Duellman and Lehr, 2009 |
| *Noblella chirihampatu* | 0 | Catenazzi and Ttito 2016 |
| *Noblella glauca* | 1 | Catenazzi and Ttito, 2018 |
| *Noblella losamigos* | 1 | Santa-Cruz et al. 2019 |
| *Noblella madreselva* | 0 | Catenazzi et al. 2015 |
| *Noblella peruviana* | 1 | von May et al. 2024 |
| *Noblella pygmaea* | 0 | Lehr and Catenazzi, 2009b |
| *Noblella thiuni* | 1 | Catenazzi and Ttito 2019 |
| *Noblella usurpator* | 0 | KU 138939, 138957, 138962 |
| *Noblella vilcabambensis* | 0 | Condori et al. 2020 |
| *Oreobates amarakaeri* | 0 | Padial et al. 2012 |
| *Oreobates antrum* | 1 | Vaz-Silva et al. 2018 |
| *Oreobates ayacucho* | 0 | Lehr 2007 |
| *Oreobates barituensis* | 0 | Vaira and Ferrari 2008 |
| *Oreobates berdemenos* | 0 | Pereyra et al. 2014 |
| *Oreoabates choristolemma* | 0 | Harvey and Sheehy 2005 |
| *Oreobates cruralis* | 0 | KU 215461, 215462 |
| *Oreobates discoidalis* | 0 | KU 182813, 182815 |
| *Oreobates gemcare* | 0 | Padial et al. 2012 |
| *Oreobates granulosus* | 0 | Duellman and Lehr, 2009 |
| *Oreobates heterodactylus* | 0 | Pansonato et al. 2020 |
| *Oreobates ibischi* | 0 | Reichle et al. 2001 |
| *Oreobates lehri* | 0 | Padial et al. 2007b |
| *Oreobates lundbergi* | 0 | Lehr 2005 |
| *Oreobates machiguenga* | 0 | Padial et al. 2012 |
| *Oreobates madidi* | 0 | Padial et al. 2005 |
| *Oreobates pereger* | 0 | KU 151909 |
| *Oreobates quixensis* | 0 | KU 126233, 178249 |
| *Oreobates remotus* | 0 | Teixeira et al. 2012 |
| *Oreobates sanctaecrucis* | 0 | Harvey and Keck 1995 |
| *Oreobates sanderi* | 0 | AMNH 6063 |
| *Oreobates saxatilis* | 0 | KU 212327, 217329 |
| *Oreobates yanucu* | 0 | Köhler and Padial. 2016 |
| *Phrynopus auriculatus* | 0 | KU 291633 |
| *Phrynopus badius* | 1 | Lehr et al. 2012 |
| *Phrynopus barthlenae* | 0 | Duellman and Lehr, 2009 |
| *Phrynopus bracki* | 0 | Hedges 1990 |
| *Phrynopus bufoides* | 0 | Duellman and Lehr, 2009 |
| *Phrynopus daemon* | 0 | Chávez et al. 2015 |
| *Phrynopus heimorum* | 0 | Duellman and Lehr, 2009 |
| *Phrynopus horstpauli* | 0 | KU 291399, 291400 |
| *Phrynopus inti* | 0 | Lehr et al. 2017a |
| *Phrynopus interstinctus* | 0 | Lehr and Oróz 2012 |
| *Phrynopus juninensis* | 0 | KU 138880, 138881 |
| *Phrynopus kauneorum* | 0 | Lehr et al. 2002 |
| *Phrynopus mariellaleo* | 0 | Venegas et al. 2008 |
| *Phrynopus miroslawae* | 0 | Duellman and Lehr, 2009 |
| *Phrynopus montium* | 0 | KU 206649, 206650 |
| *Phrynopus peruanus* | 0 | Duellman and Lehr, 2009 |
| *Phrynopus pesantesi* | 0 | Duellman and Lehr, 2009 |
| *Phrynopus tautzorum* | 0 | Duellman and Lehr 2009 |
| *Phrynopus tribulosus* | 0 | KU 291630 |
| *Phrynopus unchog* | 0 | Lehr and Rodríguez, 2017 |
| *Phrynopus vestigiatus* | 0 | Lehr and Oróz 2012 |
| *Phyllonastes cerrogolondrinas* | 1 | Ortega et al. 2025 |
| *Phyllonastes coloma* | 1 | Coloma and Duellman 2025 |
| *Phyllonastes dicaprioi* | 1 | Ortega et al. 2025 |
| *Phyllonastes ecuadoriensis* | 1 | Ortega et al. 2025 |
| *Phyllonastes heyeri* | 1 | KU 196529, 196530 |
| *Phyllonastes lynchi* | 1 | KU 212318, 212319 |
| *Phyllonastes lochites* | 1 | KU 147070 177356, Harvey et al. 2013 |
| *Phyllonastes macuma* | 1 | Ortega et al. 2025 |
| *Phyllonastes myrmecoides* | 1 | Ortega et al. 2025 |
| *Phyllonastes mindo* | 1 | Coloma and Duellman 2025 |
| *Phyllonastes naturetrekii* | 1 | Reyes-Puig et al. 2019 |
| *Phyllonastes personinus* | 1 | Coloma and Duellman 2025 |
| *Phyllonastes plateadensis* | 1 | Ortega et al. 2025 |
| *Phyllonastes sardinayacu* | 1 | Ortega et al. 2025 |
| *Phyllonastes worleyae* | 1 | Reyes-Puig et al. 2021 |
| *Pristimantis acatallelus* | 0 | ICN 7814 |
| *Pristimantis actites* | 0 | KU 141781, 141792 |
| *Pristimantis acuminatus* | 0 | KU 209467 |
| *Pristimantis boulengeri* | 0 | KU 169052, KU 169062 |
| *Pristimantis bounides* | 0 | Lehr et al. 2017b |
| *Pristimantis brevifrons* | 0 | ICN 5220, field notes |
| *Pristimantis buckleyi* | 0 | KU 217867, field notes |
| *Pristimantis chalceus* | 0 | KU 212546, 212548 |
| *Pristimantis colomai* | 1 | Lynch and Duellman 1997 |
| *Pristimantis condor* | 0 | KU 146992, 146995, 147023 |
| *Pristimantis cremnobates* | 0 | KU 166046, 166051 |
| *Pristimantis curtipes* | 0 | KU 217871, KU 130623, KU 130548 |
| *Pristimantis danae* | 0 | KU 162309, 162318 |
| *Pristimantis erythropleura* | 0 | KU 168031, 168199, field notes |
| *Pristimantis euphronides* | 1 | KU 265442, 93337 |
| *Pristimantis festae* | 0 | KU 218250 |
| *Pristimantis galdi* | 0 | KU 146980, KU 146983 |
| *Pristimantis gentry* | 0 | KU 131540, KU 131541, KU 131542 |
| *Pristimantis kelephus* | 0 | ICN 39637, field notes |
| *Pristimantis latidiscus* | 0 | KU 291199, KU 291211 |
| *Pristimantis leoni* | 0 | KU 130871, KU 130872 |
| *Pristimantis lindae* | 0 | KU 162305 |
| *Pristimantis nyctophylax* | 0 | KU 110909, KU 110926 |
| *Pristimantis ornatus* | 0 | KU 303042 |
| *Pristimantis palmeri* | 0 | ICN 29348, field notes |
| *Pristimantis pugnax* | 0 | KU 146467 |
| *Pristimantis quinquagesimus* | 0 | KU 179365 |
| *Pristimantis reichlei* | 0 | KU 207708 |
| *Pristimantis rhodoplichus* | 0 | KU 219786 |
| *Pristimantis sagittulus* | 0 | KU 291576 |
| *Pristimantis schultei* | 0 | KU 209504, KU 212224 |
| *Pristimantis simonsii* | 0 | KU 212348, 212349 |
| *Pristimantis stictogaster* | 0 | KU 291659 |
| *Pristimantis tenebrionis* | 0 | KU 179225 |
| *Pristimantis thectopternus* | 0 | KU 143938, field notes |
| *Pristimantis thymelensis* | 0 | KU 117719, 202519 |
| *Pristimantis unistrigatus* | 0 | KU 131025, 131035 |
| *Pristimantis urichi* | 0 | KU 265457, 265458 |
| *Pristimantis versicolor* | 0 | KU 119858, 119867, 119927 |
| *Pristimantis walkeri* | 0 | KU 131652, 131653 |
| *Pristimantis w-nigrum* | 0 | KU 221302, field notes |
| *Qosqophryne flammiventris* | 0 | Lehr and Catenazzi 2010 |
| *Qosqophryne gymnotis* | 0 | Lehr and Catenazzi 2009a |
| *Qosqophryne mancoinca* | 0 | Mamani et al. 2017 |
| *Strabomantis anomalus* | 0 | KU 165130, 167898, 177631 |
| *Strabomantis biporcatus* | 0 | KU 166373, 185672 |
| *Strabomantis bufoniformis* | 0 | ICN 9321,17050, 107092, 113788 |
| *Strabomantis necerus* | 0 | KU 165542, 179077, 179079 |
| *Strabomantis sulcatus* | 0 | KU 123586, 126172, 1426120 |
| *Urkuphryne merinoi* | 1 | Ortega et al. 2025 |
| *Yunganastes ashkapara* | 1 | Köhler, 2000 |
| *Yunganastes bisignatus* | 0 | Padial et al. 2007b |
| *Yunganastes fraudator* | 0 | USNM 146568 |
| *Yunganastes mercedesae* | 0 | USNM 346140, UTA 53291 |
| *Yunganastes pluvicanorus* | 0 | AMNH 165193 |

**References**

Aguayo-Vedia CR, Harvey B (2001) Dos nuevas especies de *Phrynopus* (Anura: Leptodactylidae) de los bosques nublados de Bolivia. Revista de Biología Tropical. San José 49: 333–345. https://www.scielo.sa.cr/scielo.php?pid=S0034-77442001000100033&script=sci_abstract&tlng=es

Catenazzi A, Uscapi V, von May R (2015). A new species of *Noblella* (Amphibia, Anura, Craugastoridae) from the humid montane forests of Cusco, Peru. ZooKeys 516: 71–84. https://doi.org/10.3897/zookeys.516.9776

Catenazzi A, Ttito A (2016) A new species of *Psychrophrynella* (Amphibia, Anura, Craugastoridae) from the humid montane forests of Cusco, eastern slopes of the Peruvian Andes. PeerJ 4(e1807): 1–23. http://doi.org/10.7717/peerj.1807

Catenazzi A, Ttito A, Diaz MI, Shepack A (2017) *Bryophryne phuyuhampatu* sp. n., a new species of Cusco Andes frog from the cloud forest of the eastern slopes of the Peruvian Andes (Amphibia, Anura, Craugastoridae). ZooKeys 685: 65–81. https://doi.org/10.3897/zookeys.685.12152

Catenazzi A, Ttito A (2018) *Psychrophrynella glauca* sp. n., a new species of terrestrial-breeding frogs (Amphibia, Anura, Strabomantidae) from the montane forests of the Amazonian Andes of Puno, Peru. PeerJ 4444: 1–18. https://doi.org/10.7717/peerj.4444

Catenazzi A, Ttito A (2019) *Noblella thiuni* sp. n., a new (singleton) species of minute terrestrial-breeding frog (Amphibia, Anura, Strabomantidae) from the montane forest of the Amazonian Andes of Puno, Peru. PeerJ 7(e6780): 1–15. https://peerj.com/articles/6780/

Chávez G, Santa-Cruz R, Rodríguez FD, Lehr E (2015) Two new species of frogs of the genus *Phrynopus* (Anura: Terrarana: Craugastoridae) from the Peruvian Andes. Amphibian & Reptile Conservation 9(1, e105): 15–25. https://archive.org/details/biostor-192989

Chaparro Auza JC, Padial JM, Gutiérrez RC, De la Riva I (2015) A new species of Andean frog of the genus *Bryophryne* from southern Peru (Anura: Craugastoridae) and its phylogenetic position, with notes on the diversity of the genus. Zootaxa 3994: 94–108. https://doi.org/10.11646/zootaxa.3994.1.4

Coloma LA, Duellman WE (2025). Amphibians of Ecuador. Vol. 3. Craugastoridae. Boca Raton, FL: CRC Press, 584 pp.

Condori FP, Acevedo-Rincón AA, Mamani L, Delgado AJ, Chaparro Auza JC (2020) A new species of terrestrial-breeding frog of the genus *Psychrophrynella* (Anura: Strabomantidae) from the Cordillera de Vilcabamba, southeastern Peru. Amphibian & Reptile Conservation 14 (3: e260): 127–137.

De la Riva I, Burrowes PA (2014) A new species of *Psychrophrynella* (Anura: Craugastoridae) from the Cordillera Real, Department La Paz, Bolivia . Zootaxa 3887: 459–470. https://doi.org/10.11646/zootaxa.3887.4.4).

De la Riva I (2007) Bolivian frogs of the genus *Phrynopus*, with the description of twelve new species (Anura: Brachycephalidae). Herpetological Monographs 21: 241–277. https://doi.org/10.1655/07-011.1

De la Riva I, Cortez-Fernandez C, Burrowes PA (2017) A new species of *Microkayla* (Anura: Craugastoridae: Holoadeninae) from Department La Paz, Bolivia. Zootaxa 4363: 350–360 https://doi.org/10.11646/zootaxa.4363.3.2)

De la Riva I, Chaparro Auza JS, Castroviejo-Fisher S, Padial JM (2017 "2018") Underestimated anuran radiations in the high Andes: five new species and a new genus of *Holoadeninae*, and their phylogenetic relationships (Anura: Craugastoridae). Zoological Journal of the Linnean Society 182: 129–172. https://doi.org/10.1093/zoolinnean/zlx020

Dias IR, de Mira-Mendes CV, Souza-Costa CA, Juncá FA, Solé M (2017) The advertisement call and comments on the distribution of *Eleutherodactylus bilineatus* Bokermann, 1975, an endemic frog of Bahia State, Brazil (Amphibia, Anura). ZooKeys 677: 151–159 http://dx.doi.org/10.3897/zookeys.677.12309

Duellman WE, Lehr E (2009) Terrestrial-breeding frogs (Strabomantidae) in Peru. Münster, Germany: Nature und Tier Verlag, 384 pp.

Ferreira RB, Silva-Soares T, Tonini JFR, Dantas de B (2010) New records and distribution of a species vulnerable to extinction: *Euparkerella tridactyla* (Anura, Strabomantidae). Herpetology Notes 3: 57–60.

Fusinatto LA, Alexandrino J, Haddad CFB, Brunes TO, da Rocha CFD, Sequeira F (2013) Cryptic genetic siversity is paramount in small-bodied amphibians of the genus *Euparkerella* (Anura: Craugastoridae) endemic to the Brazilian Atlantic forest. PLoS (Public Library of Science) One 8(11: e79504): 1–12. https://doi.org/10.1371/journal.pone.0079504

Harvey MB, Keck MB (1995) A new species of *Ischnocnema* (Anura: Leptodactylidae) from high elevations in the Andes of central Bolivia. Herpetologica 51: 56–66. https://www.jstor.org/stable/3892785

Harvey MB, Sheehy CM (2005) A new species of *Ischnocnema* (Anura: Leptodactylidae) from La Paz, Bolivia. Herpetologica 61: 268–275. https://doi.org/10.1655/04-71.1

Harvey MB, Almendáriz A, Brito-M J, Batallas-Revelo D (2013) A new species of *Noblella* (Anura: Craugastoridae) from the Amazonian slopes of the Ecuadorian Andes with comments on *Noblella lochites* (Lynch). Zootaxa 3635: 1–14. https://doi.org/(DOI: 10.11646/zootaxa.3635.1.1)

Hedges SB (1990) A new species of *Phrynopus* (Anura: Leptodactylidae). Copeia 1990: 108–112.

Köhler, J. 2000. New species of *Eleutherodactylus* (Anura: Leptodactylidae) from cloud forest of Bolivia. Copeia 2000: 516–520. https://doi.org/10.2307/1445826

Köhler J, Padial JM (2016) Description and phylogenetic position of a new (singleton) species of *Oreobates* Jiménez de la Espada, 1872 (Anura: Craugastoridae) from the Yungas of Cochabamba, Bolivia. Annals of the Carnegie Museum. Pittsburgh 84: 23–38. https://doi.org/10.2992/007.084.0104

Lehr E (2005) A new species of the *Eleutherodactylus nigrovittatus* group (Anura: Leptodactylidae) from Andean Peru. Herpetologica 61: 199–208. https://doi.org/10.1655/04-33

Lehr E (2006) Taxonomic status of some species of Peruvian *Phrynopus* (Anura: Leptodactylidae), with the description of a new species from the Andes of southern Peru. Herpetologica 62: 331–347. https://www.jstor.org/stable/3893490

Lehr E (2007) New eleutherodactyline frogs (Leptodactylidae: *Pristimantis*, *Phrynopus*) from Peru. Bulletin of the Museum of Comparative Zoology. Cambridge, Massachusetts 159: 145–178. https://doi.org/10.3099/0027-4100(2007)159[145:NEFLPP]2.0.CO;2

Lehr E, Aguilar C, Köhler G (2002) Two sympatric new species of *Phrynopus* (Anura: Leptodactylidae) from a cloud forest in the Peruvian Andres. Journal of Herpetology 36: 208–216. https://doi.org/10.1670/0022-1511(2002)036[0208:TSNSOP]2.0.CO;2

Lehr E, Catenazzi A (2008) A new species of *Bryophryne* (Anura: Strabomantidae) from southern Peru. Zootaxa 1784: 1–10. https://doi.org/10.11646/zootaxa.1784.1.1

Lehr E, Catenazzi A (2009a) Three new species of *Bryophryne* (Anura: Strabomantidae) from the region of Cusco, Peru. South American Journal of Herpetology 4: 125–138. https://doi.org/10.2994/057.004.0204

Lehr E, Catenazzi A (2009b) A new species of minute *Noblella* (Anura: Strabomantidae) from southern Peru: the smallest frog of the Andes. Copeia 2009: 148–156. https://doi.org/10.1643/CH-07-270

Lehr E, Catenazzi A (2010) Two new species of *Bryophryne* (Anura: Strabomantidae) from high elevations in southern Peru (region of Cusco). Herpetologica 66: 308–319. https://doi.org/10.1655/09-038.1

Lehr E, Oróz A (2012) Two new species of *Phrynopus* (Anura: Strabomantidae) from the Cordillera de Carpish in central Peru (Departamento de Huánuco). Zootaxa 3512: 53–63 https://doi.org/10.11646/zootaxa.3512.1.3

Lehr E, Rodríguez D (2017) Two new species of Andes Frogs (Craugastoridae: *Phrynopus*) from the Cordillera de Carpish in central Peru. Salamandra 53: 327–338. https://www.salamandra-journal.com/index.php/contents/2017-vol-53/1850-lehr-e-d-rodriguez

Lehr E, Moravec J, Cusi Martínez JC (2012) Two new species of *Phrynopus* (Anura, Strabomantidae) from high elevations in the Yanachaga-Chemillén National park in Peru (Departamento de Pasco). ZooKeys 235: 51–71. https://doi.org/10.3897/zookeys.235.3885

Lehr E, von May R, Moravec J, Cusi Martínez JS (2017a) A new species of *Phrynopus* (Amphibia, Anura, Craugastoridae) from upper montane forests and high Andean grasslands of the Pui Pui Protected Forest in central Peru. ZooKeys 713: 131–157. https://doi.org/10.3897/zookeys.713.20776

Lehr E, von May R, Moravec J, Cusi Martínez JC (2017b) Three new species of *Pristimantis* (Amphibia, Anura, Craugastoridae) from upper montane forests and high Andean grasslands of the Pui Pui Protected Forest in central Peru. Zootaxa 4299: 301–336 https://doi.org/10.11646/zootaxa.4299.3.1

Lynch JD (1974) A new species of leptodactylid frog (*Ischnocnema*) from the Cordillera del Condor in Ecuador. Journal of Herpetology 8: 85–87. https://www.jstor.org/stable/3893471

Lynch JD, Duellman WE (1997) Frogs of the genus *Eleutherodactylus* in western Ecuador. Systematics, ecology, and biogeography. Special Publication. Natural History Museum, University of Kansas 23: 1–236. https://archive.org/details/frogsofgenuseleu00lync

Mamani L, Catenazzi A, Ttito A, Mallqui S, Chaparro Auza JC (2017) A new species of *Bryophryne* (Anura: Strabomantidae) from the Cordillera de Vilcabamba, southeastern Peruvian Andes. Phyllomedusa. Belo Horizonte 16: 129–141. https://doi.org/10.11606/issn.2316-9079.v16i2p129-141

Motta AP, Chaparro Auza JC, Pombal JP, Guayasamin JM, De la Riva I, Padial JM (2016) Molecular phylogenetics and taxonomy of the Anean genus Lynchius Hedges, Duellman, and Heinicke, 2008 (Anura: Craugastoridae). Herpetological Monographs 30: 119–142. https://doi.org/10.1655/HERPMONOGRAPHS-D-16-00002

Ortega JA, Cisneros-Heredia DF, Camper JD, Romero-Carvajal A, Negrete L, Ron SR (2025) Systematics of minute strabomantid frogs allocated to the genus *Noblella* (Amphibia: Anura) with description of a new genus, seven new species, and insights into historical biogeography. Zoological Journal of the Linnean Society 203: 1–60. https://doi.org/10.1093/zoolinnean/zlae162 (https://doi.org/10.1093/zoolinnean/zlae162).

Padial JM, Gonzales-Álvarez L, De la Riva I (2005) A new species of the *Eleutherodactylus discoidalis* group (Anura: Leptodactylidae) from Andean humid montane forests of Bolivia. Herpetologica 61: 318–325. https://doi.org/10.1655/04-73.1

Padial JM, Castroviejo-Fisher S, Köhler J, Domic E, De la Riva I (2007b) Systematics of the *Eleutherodactylus fraudator* species group (Anura: Brachycephalidae). Herpetological Monographs 21: 213–240. https://doi.org/10.1655/06-007.1

Padial JM, Chaparro Auza JC, Castroviejo-Fisher S, Guayasamin JM, Lehr E, Delgado AJ, Vaira M, Teixeira M, Aguayo-Vedia CR, De la Riva I (2012) A revision of species diversity in the Neotropical genus *Oreobates* (Anura: Strabomantidae), with the description of three new species from the Amazonian slopes of the Andes. American Museum Novitates 3752: 1–55 http://digitallibrary.amnh.org/handle/2246/6321

Pansonato A, Motta AP, Cacciali P, Haddad CFB, Strüssmann C, Jansen M (2020) On the identity of species of *Oreobates* (Anura: Craugastoridae) from central South America, with the description of a new species from Bolivia. Journal of Herpetology 54: 393–412 http://doi.org/10.1670/20-001

Pereyra MO, Cardozo DE, Baldo JL, Baldo D (2014) Description and phylogenetic position of a new species of *Oreobates* (Anura: Craugastoridae) from northwestern Argentina. Herpetologica 70: 211–227. http://doi.org/10.1655/HERPETOLOGICA-D-13-00072

Reichle S, Lötters S, De la Riva I (2001) A new species of the *discoidalis* group of *Eleutherodactylus* (Anura, Leptodactylidae) fromi Inner-Andean dry valleys of Bolivia. Journal of Herpetology 35: 21–26. https://doi.org/10.2307/1566018

Reyes-Puig JP, Reyes-Puig C, Ron SR, Ortega JA, Guayasamin JM, Goodrum M, Recalde F, Vieira J, Koch C, Yánez-Muñoz MH (2019) A new species of terrestrial frog of the genus *Noblella* Barbour, 1930 (Amphibia: Strabomantidae) from the Llanganates-Sangay Ecological Corridor, Tungurahua, Ecuador. PeerJ 7 (e7405): 1–26. http://doi.org/10.7717/peerj.7405

Reyes-Puig C, Guayasamin JM, Koch C, Brito-Zapata D, Hollanders M, Costales M, Cisneros-Heredia DF (2021) A new species of the genus *Noblella* (Amphibia: Strabomantidae) from Ecuador, with new information for *Noblella worleyae*. Acta Herpetologica. Firenze 16: 63-87. http://doi.org/10.36253/a_h-10742

Reyes-Puig JP, Yánez-Muñoz MH, Urgilés-Merchán MA, Libke Z, Ron SR, Carrión-Olmedo JC (2026) Two new terrestrial narrow-toed frogs (*Niceforonia*: Strabomantidae, Anura) from remote unexplored mountains in the Ecuadorian Andes. PeerJ 14(e21182): 1–30. https://doi.org/10.7717/peerj.21182

Sánchez-Nivicela JC, Urgilés VL, Navarrete Méndez MJ, Yánez-Muñoz MH, Ron SR (2019) A bizarre new species of Lynchius (Amphibia, Anura, Strabomantidae) from the Andes of Ecuador and first report of Lynchius parkeri in Ecuador. Zootaxa 4567: 1–24. http://doi.org/10.11646/zootaxa.4567.1.1

Santa-Cruz Farfan R, von May R, Catenazzi A, Whitcher C, López Tejeda E, Rabosky DL (2019) A new species of terrestrial-breeding frog (Amphibia, Strabomantidae, *Noblella*) from the Upper Madre De Dios watershed, Amazonian Andes and lowlands of southern Peru. Diversity 11 (145): 1–20. https://doi.org/10.3390/d11090145

Teixeira M, Amaro RC, Recoder RS, de Sena MA, Rodrigues MT (2012) A relict new species of *Oreobates* (Anura, Strabomantidae) from the seasonally dry tropical forests of Minas Gerais, Brazil, and its implication to the biogeography of the genus and that of South American dry forests. Zootaxa 3158: 37–52. https://doi.org/10.11646/zootaxa.3158.1.3

Vaira, M, Ferrari L (2008) A new species of *Oreobates* (Anura: Strabomantidae) from the Andes of northern Argentina. Zootaxa 1908: 41–50. https://doi.org/10.11646/zootaxa.1908.1.3

Vaz-Silva W, Maciel NM, de Andrade SP, Amaro RC (2018) A new cryptic species of *Oreobates* (Anura: Craugastoridae) from the seasonally dry tropical forest of central Brazil. Zootaxa 4441: 89–108. https://doi.org/10.11646/zootaxa.4441.1.5

Venegas PJ, Barboza AC, De la Riva I, Padial JM (2018) A new species of *Phrynopus* from the northeastern Andes of Peru, its phylogenetic position, and notes on the relationships of Holoadeninae (Anura: Craugastoridae). Zootaxa 4446: 501–524. https://doi.org/10.11646/zootaxa.4446.4.5).

von May R, Diaz MI, Ttito A, anta-Cruz Farfan R, Catenazzi A (2024) The rediscovery of *Noblella peruviana* after more than 115 years helps resolve the molecular phylogeny and taxonomy of Noblella (Amphibia, Anura, Strabomantidae). Diversity 16 (613): 1–15 https://doi.org/10.3390/d16100613

Vrcibradic D, Almeida-Gomes M, Van Sluys M, da Rocha CFD (2008) Amphibia, Anura, *Hylodes charadranaetes*, *Ischnocnema octavioi*, and *Euparkerella cochranae*: distribution extension. Check List. A Journal of Species Lists and Distribution 4: 103–106. https://doi.org/10.15560/4.2.103
