# Supplementary material for: A new species of Niceforonia (Amphibia, Anura, Strabomantidae) from the Serranía de los Paraguas, Colombia, with the description of a new phenotypic synapomorphy for the Niceforonia nigrovittata species group
Source: Zookeys. 2026 Aug 11;1289:159–84. doi: 10.3897/zookeys.1289.198411 (PMC13487511; doi:10.3897/zookeys.1289.198411)
Supplement: Supplementary file 4 [file zookeys-1289-159_article-198411__-s002.docx]

| Species | Voucher | 12S | 16S | RAG1 | TYR | **References** |
| --- | --- | --- | --- | --- | --- | --- |
| *Bahius bilineatus* | CFBH32419 | MW201164 | | MW201171 | MW203021 | Motta *et al.* (2021) |
| *Barycholos pulcher* | KU217781 | EU186727 | EU186709 | EU186744 | EU186765 | Hedges *et al.* (2008) |
| *Barycholos ternetzi* | CFBH11598 | MW202395 | MW202390 | MW201168 | MW203018 | Motta *et al.* (2021) |
| *Bryophryne bakersfield* | MUBI6022 | MF186284 | MF186341 | MF186528 | - | De la Riva *et al.* (2017 "2018") |
| *Bryophryne bustamantei* | MUBI6019 | MF186296 | MF186356 | MF186544 | MF186548 | De la Riva *et al.* (2017 "2018") |
| *Bryophryne cophites* | KU173497 | EF493537 | | EF493423 | EF493508 | Heinicke *et al.* (2007) |
| *Bryophryne hanssaueri* | MUSM27567 | - | KY652642 | KY681084 | KY681063 | von May *et al*. (2017) |
| *Bryophryne nubilosus* | MUSM27882 | - | KY652643 | KY681085 | KY681064 | von May *et al*. (2017) |
| *Bryophryne phuyuhampatu* | CORBIDI18224 | - | MF419254 | - | - | Catenazzi *et al.* (2017) |
| *Bryophryne quellokunka* | MNCN43780 | MF186309 | MF186387 | MF186526 | - | De la Riva *et al.* (2017 "2018") |
| *Bryophryne tocra* | MNCN43786 | MF186315 | MF186396 | MF186541 | MF186583 | De la Riva *et al.* (2017 "2018") |
| *Bryophryne wilakunka* | MUBI5425 | MF186291 | MF186349 | - | - | De la Riva *et al.* (2017 "2018") |
| *Bryophryne zonalis* | MUSM 27939 | MT437064 | - | - | - | Catenazzi *et al.* (2020) |
| *Craugastor longirostris* | KU177803 | EF493395 | | EF493454 | EF493482 | Heinicke *et al.* (2007) |
| *Euparkerella brasiliensis* | Eubra | JX298276 | JX298316 | - | JX298237 | Fouquet et al. (2012) |
| *Euparkerella cochranae* | MNRJ56146 | MW202392 | MW202384 | MW201175 | KF625114 | Motta *et al.* (2021); Fusinatto *et al.* (2013) |
| *Euparkerella tridactyla* | JFT305 | MW202394 | MW202387 | MW201173 | KF625109 | Motta *et al.* (2021); Fusinatto *et al.* (2013) |
| *Holoaden bradei* | USNM207945 | EF493378 | EF493366 | EF493449 | EU186779 | Heinicke *et al.* (2007); Hedges et al. (2008) |
| *Holoaden luederwaldti* | MZUSP131872 | EU186728 | EU186710 | EU186747 | EU186768 | Hedges *et al.* (2008) |
| *Lynchius flavomaculatus* | KU218210 | EU186667 | | EU186745 | EU186766 | Hedges *et al.* (2008) |
| *Lynchius megacephalus* | MZUA633 | - | MK423938 | - | - | Sánchez-Nivicela *et al.* (2019) |
| *Lynchius nebulanastes* | KU181408 | EU186704 | | - | - | Hedges *et al.* (2008) |
| *Lynchius oblitus* | MHNC8677 | KX470779 | KX470786 | KX470795 | KX470802 | Motta *et al.* (2016) |
| *Lynchius parkeri* | KU181307 | EU186705 | | - | - | Hedges *et al.* (2008) |
| *Lynchius simmonsi* | QZ41639 | JF809940 | JF810004 | - | - | Padial *et al.* (2012) |
| *Lynchius tabaconas* | MHNC8650 | KX470774 | KX470781 | KX470790 | KX470797 | Motta *et al.* (2016) |
| *Microkayla adenopleura* | MNCN44810 | MF186283 | MF186340 | MF186537 | MF186565 | De la Riva *et al.* (2017 "2018") |
| *Microkayla ankohuma* | MNKA7280 | MF186288 | MF186346 | - | MF186560 | De la Riva *et al.* (2017 "2018") |
| *Microkayla boettgeri* | MUBI5363 | MF186294 | MF186353 | - | MF186559 | De la Riva *et al.* (2017 "2018") |
| *Microkayla chacaltaya* | MNCN42052 | - | MF186357 | MF186532 | - | De la Riva *et al.* (2017 "2018") |
| *Microkayla chapi* | MNCN43762 | MF186328 | MF186417 | MF186540 | MF186562 | De la Riva *et al.* (2017 "2018") |
| *Microkayla chilina* | MNCN43772 | MF186327 | MF186414 | MF186539 | MF186561 | De la Riva *et al.* (2017 "2018") |
| *Microkayla condoriri* | CBF5989 | MF186300 | MF186360 | MF186530 | MF186550 | De la Riva *et al.* (2017 "2018") |
| *Microkayla guillei* | AMNHA165108 | AY843720 | | - | DQ282995 | Faivovich *et al.* (2005); Frost *et al.* (2006) |
| *Microkayla huayna* | CBF6564 | MF186317 | MF186399 | - | MF186556 | De la Riva *et al.* (2017 "2018") |
| *Microkayla iatamasi* | MNCN42054 | MF186304 | MF186368 | MF186536 | MF186558 | De la Riva *et al.* (2017 "2018") |
| *Microkayla illampu* | CBF5998 | MF186305 | MF186369 | MF186534 | MF186549 | De la Riva *et al.* (2017 "2018") |
| *Microkayla illimani* | MNCN42071 | - | MF186374 | - | - | De la Riva *et al.* (2017 "2018") |
| *Microkayla kallawaya* | MNCN42061 | MF186306 | MF186376 | - | MF186575 | De la Riva *et al.* (2017 "2018") |
| *Microkayla katantika* | CBF6013 | MF186307 | MF186381 | MF186533 | MF186576 | De la Riva *et al.* (2017 "2018") |
| *Microkayla kempffi* | MNCN43646 | MF186308 | MF186384 | MF186538 | MF186566 | De la Riva *et al.* (2017 "2018") |
| *Microkayla quimsacruzis* | MNCN42063 | MF186323 | MF186405 | - | - | De la Riva *et al.* (2017 "2018") |
| *Microkayla saltator* | CBF6033 | MF186326 | MF186410 | - | - | De la Riva *et al.* (2017 "2018") |
| *Microkayla teqta* | MNCN45704 | MF186321 | MF186403 | - | MF186555 | De la Riva *et al.* (2017 "2018") |
| *Microkayla wettsteini* | KU183049 | EU186696 | | - | - | Hedges *et al.* (2008) |
| *Niceforonia brunnea* | KU178258 | EF493357 | | EF493422 | EF493484 | Heinicke *et al.* (2007) |
| *Niceforonia dracula* | DHMECN19407 | PX596795 | | - | - | Reyes-Puig *et al.* (2026) |
| *Niceforonia dracula* | DHMECN19408 | PX596796 | | - | - | Reyes-Puig *et al.* (2026) |
| *Niceforonia elassodiscus* | KU177282 | EF493358 | | - | - | Heinicke *et al.* (2007) |
| ***Niceforonia mantipus*** | **ARUQ-1880** | **-** | **PZ348889** | **-** | **-** | **This study** |
| ***Niceforonia mantipus*** | **MAR1275** | **PZ352892** | | **-** | **-** | **This study** |
| ***Niceforonia mantipus*** | **CD-UV (JCA 0832)** | **PZ352893** | | **-** | **-** | **This study** |
| *Niceforonia nana* | IAvHAm13055 | MH532903 | MH536810 | - | MH542227 | Acosta-Galvis *et al.* (2018) |
| *Niceforonia nigrovittata* | QCAZ33258 | PP536448 | - | - | - | Ortega *et al*. (2025) |
| *Niceforonia nigrovittata* – Clade A | DHMECN19274 | PX625817 | | - | - | Reyes-Puig *et al.* (2026) |
| *Niceforonia nigrovittata* – Clade A | DHMECN19347 | PX625818 | |  |  | Reyes-Puig *et al.* (2026) |
| *Niceforonia nigrovittata* – Clade B | CORBIDI9547 | - | | MH538300 | - | Reyes-Puig *et al.* (2026) |
| *Niceforonia nigrovittata* – Clade C | DHMECN20003 | PX625827 | | - | - | Reyes-Puig *et al.* (2026) |
| *Niceforonia nigrovittata* – Clade D | DHMECN19995 | PX625826 | | - | - | Reyes-Puig *et al.* (2026) |
| *Niceforonia nigrovittata* – Clade D | DHMECN19273 | PX625816 | | - | - | Reyes-Puig *et al.* (2026) |
| *Niceforonia peraccai* | KU178266 | EF493710 | | EF493420 | EF493485 | Heinicke *et al.* (2007) |
| *Niceforonia peraccai* | QCAZA57536 | - | PX625835 | - | - | Reyes-Puig *et al.* (2026) |
| *Niceforonia philippi* | JDL17574 | EF493394 | | EF493414 | EF493483 | Heinicke *et al.* (2007) |
| *Niceforonia quilloturo* | DHMECN19885 | PX625824 | | - | - | Reyes-Puig *et al.* (2026) |
| *Niceforonia quilloturo* | DHMECN19886 | PX625825 | | - | - | Reyes-Puig *et al.* (2026) |
| *Niceforonia* sp 1 | QCAZ41125 | - | MT372720 | MT372595 | - | Reyes-Puig *et al.* (2026) |
| *Niceforonia* sp 1 | QCAZA41046 | - | PX625829 | - | - | Reyes-Puig *et al.* (2026) |
| *Niceforonia* sp 1 | QCAZ41145 | - | PP536450 | - | - | Reyes-Puig *et al.* (2026) |
| *Niceforonia* sp 2 | QCAZ52495 | MW567328 | MW567375 | MW451754 | - | Reyes-Puig *et al.* (2026) |
| *Niceforonia* sp 2 | QCAZA52497 | - | PX625834 | - | - | Reyes-Puig *et al.* (2026) |
| *Niceforonia* sp 3 | QCAZA68992 | - | PX625837 | - | - | Reyes-Puig *et al.* (2026) |
| *Niceforonia* sp 4 | QCAZA41299 | - | PX625833 | - | - | Reyes-Puig *et al.* (2026) |
| ***Niceforonia tempestuosa* sp nov** | **ARUQ-1882** | **-** | **PZ348890** | **-** | **-** | **This study** |
| ***Niceforonia tempestuosa* sp nov** | **ARUQ-1884** | **-** | **PZ348891** | **-** | **-** | **This study** |
| *Noblella bagrecito* | CORBIDI17528 | - | PQ362287 | - | - | von May *et al.* (2024) |
| *Noblella chirihampatu* | CORBIDI1649 | - | KU884559 | - | - | Catenazzi and Ttito (2016) |
| *Noblella glauca* | CORBIDI1872 | - | MG837565 | - | - | Catenazzi and Ttito (2018) |
| *Noblella losamigos* | RvM312 | - | KY652644 | KY672962 | KY681065 | von May *et al*. (2017) |
| *Noblella madreselva* | CORBIDI1576 | - | MN064565 | MN355547 | - | Santa-Cruz Farfan *et al.* (2019); Catenazzi *et al.* (2015) |
| *Noblella peruviana* | CORBIDI1870 | - | PQ362295 | PQ374247 | PQ374260 | von May *et al.* (2024) |
| *Noblella pygmaea* | MUSM24536 | - | KY652645 | KY681086 | KY681066 | von May *et al*. (2017) |
| *Noblella thiuni* | CORBIDI18723 | - | MK072732 | - | - | Catenazzi and Ttito (2019) |
| *Noblella usurpator* | KU173495 | EF493714 | | EU186762 | EU186780 | Heinicke *et al.* (2007); Hedges et al. (2008) |
| *Noblella vilcabambensis* | MUSM27619 | - | MT437065 | - | - | Catenazzi *et al.* (2020) |
| *Oreobates amarakaeri* | MHNC6975 | JF809934 | JF809996 | JF809913 | JF809891 | Padial *et al.* (2012) |
| *Oreobates antrum* | ZUFG5890 | MH025429 | MH025453 | MH025438 | MH025447 | Vaz-Silva *et al.* (2018) |
| *Oreobates ayacucho* | MNCN(IDlR5024) | JF809933 | JF809970 | JF809912 | JF809890 | Padial *et al.* (2012) |
| *Oreobates barituensis* | MCN1359 | JF809935 | JF809999 | JF809914 | JF809892 | Padial *et al.* (2012) |
| *Oreobates berdemenos* | LGE 2530 | KJ125507 | - | - | - | Pereyra *et al* (2014) |
| *Oreobates choristolemma* | CBG765 | JF809921 | FJ539067 | JF809900 | JF809881 | Padial *et al.* (2009); Padial et al. (2012) |
| *Oreobates cruralis* | KU215462 | EU186666 | | EU186743 | EU186764 | Hedges *et al.* (2008) |
| *Oreobates discoidalis* | MNCN43133 | JF809925 | FJ539068 | JF809904 | JF809884 | Padial *et al.* (2009a); Padial et al. (2012) |
| *Oreobates gemcare* | MHNC6687 | JF809930 | JF809960 | JF809909 | - | Padial et al. (2012) |
| *Oreobates granulosus* | MHNC3396 | JF809929 | EU368897 | JF809908 | JF809887 | Padial *et al.* (2008); Padial et al. (2012) |
| *Oreobates heterodactylus* | MNKA7175 | JF809923 | EU192296 | JF809902 | JF809882 | Padial *et al.* (2009b); Padial *et al.* (2012) |
| *Oreobates ibischi* | MNKA6612 | FJ438817 | FJ438806 | - | - | Padial *et al.* (2009a) |
| *Oreobates lehri* | MUSM27616 | JF809927 | JF809957 | JF809906 | - | Padial *et al.* (2012) |
| *Oreobates lundbergi* | MTD45902 | JF809928 | JF809958 | JF809907 | JF809886 | Padial *et al.* (2012) |
| *Oreobates machiguenga* | MHNC6809 | JF809932 | JF809969 | JF809911 | JF809889 | Padial *et al.* (2012) |
| *Oreobates madidi* | MNKA7856 | JF809922 | FJ539070 | JF809901 | - | Padial *et al.* (2008); Padial et al. (2012) |
| *Oreobates pereger* | MTD46808 | JF809926 | JF809955 | JF809905 | JF809885 | Padial *et al.* (2012) |
| *Oreobates quixensis* | KU178249 | EF493828 | EF493662 | - | - | Heinicke *et al.* (2007) |
| *Oreobates remotus* | MZUSP141708 | MH025425 | MH025449 | MH025434 | MH025443 | Vaz-Silva *et al.* (2018) |
| *Oreobates sanctaecrucis* | MNKA5507 | JF809924 | JF809951 | JF809903 | JF809883 | Padial *et al.* (2012) |
| *Oreobates sanderi* | MNCN42017 | - | EU368904 | - | - | Padial *et al.* (2008) |
| *Oreobates saxatilis* | MHNC6775 | JF809931 | JF809962 | JF809910 | JF809888 | Padial *et al.* (2012) |
| *Oreobates yanucu* | ZFMK72569 | - | KY111322 | - | - | Köhler and Padial (2016) |
| *Phrynopus auriculatus* | MUBI6471 | MF186290 | MF186348 | - | MF186582 | De la Riva *et al.* (2017 "2018") |
| *Phrynopus badius* | FMNH282818 | MG896594 | MG896571 | MG896618 | - | von May *et al.* (2018) |
| *Phrynopus barthlenae* | MHNSM20609 | MF186292 | MF186350 | - | - | De la Riva *et al.* (2017 "2018") |
| *Phrynopus bracki* | USNM286919 | EF493709 | | EF493421 | EF493507 | Heinicke *et al.* (2007) |
| *Phrynopus bufoides* | MHNSM19860 | AM039713 | AM039645 | - | - | Lehr *et al.* (2005) |
| *Phrynopus daemon* | MUSM32747 | MG896597 | MG896574 | - | - | von May *et al.* (2018) |
| *Phrynopus heimorum* | MTD45621 | MF186302 | MF186363 | MF186545 | MF186580 | De la Riva *et al.* (2017 "2018") |
| *Phrynopus horstpauli* | MTD44335 | MF186303 | MF186364 | - | MF186584 | De la Riva *et al.* (2017 "2018") |
| *Phrynopus interstinctus* | MUSM29543 | MG896598 | MG896575 | MG896621 | - | von May *et al.* (2018) |
| *Phrynopus inti* | UMMZ245218 | MF651913 | MF651906 | MF651918 | MF651921 | Lehr *et al*. (2017) |
| *Phrynopus juninensis* | MUSM38323 | MG896599 | MG896576 | MG896622 | - | von May *et al.* (2018) |
| *Phrynopus kauneorum* | MTD44332 | AM039718 | AM039650 | - | - | Lehr *et al.* (2005) |
| *Phrynopus mariellaleo* | CORBIDI11669 | - | MH538297 | MH538301 | - | Venegas *et al.* (2018) |
| *Phrynopus miroslawae* | MUBI6469 | MF186312 | MF186393 | MF186542 | MF186585 | De la Riva *et al.* (2017 "2018") |
| *Phrynopus montium* | MUSM33259 | MG896601 | MG896578 | MG896624 | - | von May *et al.* (2018) |
| *Phrynopus peruanus* | MUSM38316 | MG896605 | MG896582 | MG896626 | MG896631 | von May *et al.* (2018) |
| *Phrynopus pesantesi* | MTD45072 | AM039724 | AM039656 | - | - | Lehr *et al.* (2005) |
| *Phrynopus tautzorum* | MHNSM20613 | AM039720 | AM039652 | - | - | Lehr *et al.* (2005) |
| *Phrynopus tribulosus* | MUBI6441 | MF186313 | MF186394 | MF186546 | MF186577 | De la Riva *et al.* (2017 "2018") |
| *Phrynopus unchog* | MUSM32748 | MG896608 | MG896591 | - | - | von May *et al.* (2018) |
| *Phrynopus vestigiatus* | MUSM29542 | MG896610 | MG896593 | - | - | von May *et al.* (2018) |
| *Phyllonastes cerrogolondrinas* | QCAZ66089 | - | PP536471 | PP536432 | PP536402 | Ortega et al. (2025) |
| *Phyllonastes coloma* | QCAZ40579 | - | MT710932 | - | - | Reyes-Puig *et al.* (2020) |
| *Phyllonastes dicaprioi* | QCAZ72212 | PP536488 | PP536476 | PP536437 | PP536407 | Ortega *et al.* (2025) |
| *Phyllonastes ecuadoriensis* | QCAZ4891 | - | PP536454 | PP536419 | PP536392 | Ortega *et al.* (2025) |
| *Phyllonastes heyeri* | QCAZ31471 | JX267463 | JX267541 | PP536414 | PP536388 | Canedo and Haddad (2012): Ortega *et al.* (2025) |
| *Phyllonaste lynchi* | MUSA3855 | - | PQ362307 | PQ374257 | - | von May *et al.* (2024) |
| *Phyllonastes lochites* | KU177356 | EU186699 | | EU186756 | EU186777 | Hedges *et al.* (2008) |
| *Phyllonastes macuma* | QCAZ40180 | JX267464 | JX267542 | PP536415 | PP536389 | Canedo and Haddad (2012): Ortega *et al.* (2025) |
| *Phyllonastes mindo* | ZSFQ051 | - | OM200088 | - | - | Reyes-Puig *et al.* (2021) |
| *Phyllonastes myrmecoides* | QCAZ53583 | - | PP536458 | PP536421 | PP536394 | Ortega *et al.* (2025) |
| *Phyllonastes naturetrekii* | QCAZ71337 | MK838467 | MK838462 | PP536434 | PP536404 | Reyes-Puig *et al.* (2019); Ortega *et al.* (2025) |
| *Phyllonastes personinus* | QCAZ58818 | MK838468 | MK838465 | MN068028 | - | Reyes-Puig *et al.* (2019) |
| *Phyllonastes plateadensis* | QCAZ65015 | - | PP536467 | PP536428 | PP536398 | Ortega *et al.* (2025) |
| *Phyllonastes sardinayacu* | QCAZ58822 | PP536498 | PP536463 | PP536426 | PP536396 | Ortega *et al.* (2025) |
| *Phyllonastes worleyae* | ZSFQ550 | - | MT710934 | - | - | Reyes-Puig *et al.* (2020) |
| *Pristimantis_acatallelus* | UVC15914 | JN104675 | - | - | KT898368 | García-R *et al.* (2012); Mendoza *et al.* (2015) |
| *Pristimantis actites* | KU217830 | EF493696 | | EF493432 | EF493494 | Heinicke *et al.* (2007) |
| *Pristimantis acuminatus* | QCAZ53263 | MN128394 | - | - | - | Ortega-Andrade *et al.* (2015) |
| *Pristimantis boulengeri* | ICN55788 | KY494232 | | KY494205 | KY494217 | Gonzalez-Duran *et al.* (2017) |
| *Pristimantis bounides* | MUSM31198 | - | KY962794 | KY962771 | - | Lehr *et al.* (2017) |
| *Pristimantis brevifrons* | UVC15858 | - | JN370956 | - | KT898342 | García-R *et al.* (2012); Mendoza *et al.* (2015) |
| *Pristimantis buckleyi* | KU217836 | EF493350 | |  |  | Heinicke *et al.* (2007) |
| *Pristimantis chalceus* | KU177638 | EF493675 | | - | - | Heinicke *et al.* (2007) |
| *Pristimantis colomai* | QCAZ17101 | EF493354 | | EF493440 | EF493502 | Heinicke *et al.* (2007) |
| *Pristimantis condor* | KU217857 | EF493701 | | EF493443 | EF493504 | Heinicke *et al.* (2007) |
| *Pristimantis cremnobates* | KU177252 | EF493528 | | EF493424 | EF493486 | Heinicke *et al.* (2007) |
| *Pristimantis curtipes* | KU217871 | EF493513 | | EF493435 | EF493497 | Heinicke *et al.* (2007) |
| *Pristimantis danae* | MVZ272358 | - | KY652652 | KY672968 | KY681073 | von May *et al.* (2017) |
| *Pristimantis erythropleura* | UVC15886 | - | JN371036 | KT898309 | KT898348 | García-R *et al.* (2012); Mendoza *et al.* (2015) |
| *Pristimantis euphronides* | BWMC6918 | EF493527 | | EF493427 | EF493489 | Heinicke *et al.* (2007) |
| *Pristimantis festae* | KU218234 | EF493515 | |  |  | Heinicke *et al.* (2007) |
| *Pristimantis galdi* | QCAZ32368 | EU186670 | | EU186746 | EU186767 | Heinicke *et al.* (2007); Hedges *et al*. (2008) |
| *Pristimanti gentryi* | KU218109 | EF493511 | | - | - | Heinicke et al. (2007) |
| *Pristimantis kelephus* | UVC15870 | - | JN104660 | KT898319 | KT898357 | García-R *et al.* (2014); Mendoza *et al.* (2015) |
| *Pristimantis latidiscus* | KU218016 | EF493698 | | - | - | Heinicke *et al.* (2007) |
| *Pristimantis leoni* | KU218227 | EF493684 | | EF493433 | EF493495 | Heinicke *et al. (*2007) |
| *Pristimantis lindae* | MUSM27902 | - | KY652653 | KY672969 | KY681074 | von May *et al.* (2017) |
| *Pristimantis nyctophylax* | KU177812 | EF493526 | | EF493425 | EF493487 | Heinicke *et al.* (2007) |
| *Pristimantis ornatus* | MTD45073 | EU186660 | | - | - | Hedges *et al.* (2008) |
| *Pristimantis palmeri* | UVC15823 | - | JN371003 | KT898326 | KT898364 | García-R *et al.* (2012); Mendoza *et al.* (2015) |
| *Pristimantis pugnax* | KU202432 | AY326005 | | - | - | Darst and Cannatella (2004) |
| *Pristimantis quinquagesimus* | KU179374 | EF493690 | | - | - | Heinicke *et al.* (2007) |
| *Pristimantis reichlei* | MHNSM9267 | EF493707 | | EF493436 | EF493498 | Heinicke *et al.* (2007) |
| *Pristimantis rhodoplichus* | KU219788 | EF493674 | | - | - | Heinicke *et al.* (2007) |
| *Pristimantis sagittulus* | KU291635 | EF493705 | | EF493439 | EF493501 | Heinicke *et al.* (2007) |
| *Pristimantis schultei* | KU212220 | EF493681 | | - | - | Heinicke *et al.* (2007) |
| *Pristimantis simonsii* | KU212350 | EU186665 | | - | - | Hedges *et al.* (2008) |
| *Pristimantis stictogaster* | KU291659 | EF493704 | | EF493445 | EF493506 | Heinicke *et al.* (2007) |
| *Pristimantis tenebrionis* | QCAZ65582 | - | MT372685 | MT372631 | - | Reyes-Puig C. *et al.* (2020) |
| *Pristimantis thectopternus* | UVC15954 | - | JN104685 | KT898330 | KT898369 | García-R *et al.* (2014); Mendoza *et al.* (2015) |
| *Pristimantis thymelensis* | TNHCGDC14370 | JX564889 | | - | - | Zhang *et al.* (2013) |
| *Pristimantis unistrigatus* | KU218057 | EF493387 | | EF493444 | EF493505 | Heinicke *et al.* (2007) |
| *Pristimantis urichi* | USNM336098 | EF493699 | | EF493426 | EF493488 | Heinicke *et al.* (2007) |
| *Pristimantis versicolor* | KU218096 | EF493389 | | EF493431 | EF493493 | Heinicke *et al.* (2007) |
| *Pristimantis walkeri* | KU218116 | EF493518 | | EF493428 | EF493490 | Heinicke *et al.* (2007) |
| *Pristimantis w-nigrum* | UVC15923 | - | JN104686 | KT898311 | KT898370 | García-R *et al.* (2014); Mendoza *et al.* (2015) |
| *Qosqophryne flammiventris* | MUSM27615 | - | MT437056 | MT431913 | - | Catenazzi *et al.* (2020) |
| *Qosqophryne gymnotis* | MUSM24546 | - | MT437057 | MT431914 | - | Catenazzi *et al.* (2020) |
| *Qosqophryne mancoinca* | MUBI16068 | - | MT437059 | - | - | Catenazzi *et al.* (2020) |
| *Strabomantis anomalus* | KU177627 | EF493534 | | EF493447 | - | Heinicke *et al.* (2007) |
| *Strabomantis biporcatus* | CVULA7073 | EU186691 | | EU186754 | EU186775 | Heinicke *et al.* (2007); Hedges *et al.* (2008) |
| *Strabomantis bufoniformis* | SIUC7062 | DQ283165 | | - | DQ282942 | Frost *et al.* (2006) |
| *Strabomantis necerus* | KU179076 | EF493535 | | - | - | Heinicke *et al.* (2007) |
| *Strabomantis sulcatus* | KU218055 | EF493536 | | - | - | Heinicke *et al.* (2007) |
| *Urkuphryne merinoi* | QCAZ66091 | PP536500 | PP536473 | PP536433 | PP536403 | Ortega *et al.* (2025) |
| *Yunganastes ashkapara* | ZFMK70318 | FJ438807 | FJ438796 | JF809919 | JF809898 | Padial *et al.* (2009); Padial *et al.* (2012) |
| *Yunganastes bisignatus* | MNCNDNA6331 | FJ438808 | FJ438797 | - | - | Padial *et al.* (2009a) |
| *Yunganastes fraudator* | MNCN43107 | JF809938 | FJ539065 | JF809916 | JF809895 | Padial *et al.* (2009); Padial *et al.* (2012) |
| *Yunganastes mercedesae* | ZFMK72572 | FJ539071 | FJ539066 | - | - | Padial *et al.* (2009a) |
| *Yunganastes pluvicanorus* | MNCNDNA6004 | JF809937 | EU192247 | JF809917 | JF809896 | Padial et al. (2008); Padial *et al.* (2009); Padial *et al.* (2012) |

**References**

Acosta-Galvis, A. R, Streicher JW, Manuelli L, Cuddy T, de Sá RO (2018) Molecular insights into the phylogenetic placement of the poorly known genus *Niceforonia* Goin and Cochran, 1963 (Anura: Brachycephaloidea). Zootaxa 4514: 487–500.

Canedo, C. and Haddad, C.F (2012) Phylogenetic relationships within anuran clade Terrarana, with emphasis on the placement of Brazilian Atlantic rainforest frogs genus *Ischnocnema* (Anura: Brachycephalidae). Molecular Phylogenetics and evolution, 65(2): 610–620.

Catenazzi A, Ttito A (2016) A new species of *Psychrophrynella* (Amphibia, Anura, Craugastoridae) from the humid montane forests of Cusco, eastern slopes of the Peruvian Andes. PeerJ 4(e1807): 1–23. http://doi.org/10.7717/peerj.1807

Catenazzi A, Ttito A (2018) *Psychrophrynella glauca* sp. n., a new species of terrestrial-breeding frogs (Amphibia, Anura, Strabomantidae) from the montane forests of the Amazonian Andes of Puno, Peru. PeerJ 4444: 1–18. https://doi.org/10.7717/peerj.4444

Catenazzi A, Ttito A (2019) *Noblella thiuni* sp. n., a new (singleton) species of minute terrestrial-breeding frog (Amphibia, Anura, Strabomantidae) from the montane forest of the Amazonian Andes of Puno, Peru. PeerJ 7(e6780): 1–15. https://peerj.com/articles/6780/

Catenazzi A, Ttito A, Diaz MI, Shepack A (2017) *Bryophryne phuyuhampatu* sp. n., a new species of Cusco Andes frog from the cloud forest of the eastern slopes of the Peruvian Andes (Amphibia, Anura, Craugastoridae). ZooKeys 685: 65–81.

Catenazzi A, Uscapi V, von May R (2015). A new species of *Noblella* (Amphibia, Anura, Craugastoridae) from the humid montane forests of Cusco, Peru. ZooKeys 516: 71–84. https://doi.org/10.3897/zookeys.516.9776

Catenazzi, A., L. Mamani, E. Lehr, and R. von May (2020) A new genus of terrestrial-breeding frogs (Holoadeninae, Strabomantidae, Terrarana) from southern Peru. Diversity 12 (184): 1–17. http://doi.org//10.3390/d12050184

Darst, C.R. and Cannatella, D.C (2004) Novel relationships among hyloid frogs inferred from 12S and 16S mitochondrial DNA sequences. Molecular phylogenetics and evolution, 31(2): 462–475.

De la Riva I, Chaparro Auza JS, Castroviejo-Fisher S, Padial JM (2017 "2018") Underestimated anuran radiations in the high Andes: five new species and a new genus of Holoadeninae, and their phylogenetic relationships (Anura: Craugastoridae). Zoological Journal of the Linnean Society 182: 129–172. https://doi.org/10.1093/zoolinnean/zlx020

Faivovich, J., C. F. B. Haddad, P. C. de A. Garcia, D. R. Frost, J. A. Campbell, and W. C. Wheeler (2005) Systematic review of the frog family Hylidae, with special reference to Hylinae: A phylogenetic analysis and taxonomic revision. Bulletin of the American Museum of Natural History 294: 1–240.

Fouquet, A., Loebmann, D., Castroviejo-Fisher, S., Padial, J.M., Orrico, V.G., Lyra, M.L., Roberto, I.J., Kok, P.J., Haddad, C.F. and Rodrigues, M.T (2012) From Amazonia to the Atlantic forest: Molecular phylogeny of Phyzelaphryninae frogs reveals unexpected diversity and a striking biogeographic pattern emphasizing conservation challenges. Molecular phylogenetics and evolution, 65(2): 547–561.

Frost, D.R., Grant, T., Faivovich, J., Bain, R.H., Haas, A., Haddad, C.F., De Sa, R.O., Channing, A.L.A.N., Wilkinson, M.A.R.K., Donnellan, S.C. and Raxworthy, C.J (2006) The amphibian tree of life. Bulletin of the American Museum of natural History, 2006(297): 1–291.

Fusinatto, L.A., Alexandrino, J., Haddad, C.F., Brunes, T.O., Rocha, C.F. and Sequeira, F (2013) Cryptic genetic diversity is paramount in small-bodied amphibians of the genus *Euparkerella* (Anura: Craugastoridae) endemic to the Brazilian Atlantic Forest. PLoS one, 8(11), p.e79504.

García-R, J.C., Crawford, A.J., Mendoza, Á.M., Ospina, O., Cardenas, H. and Castro, F (2012) Comparative phylogeography of direct-developing frogs (Anura: Craugastoridae: *Pristimantis*) in the southern Andes of Colombia. PLoS ONE 7(9): e46077. https://doi.org/10.1371/journal.pone.0046077

Garcia-R, J.C., Mendoza, Á.M., Ospina, O., Cardenas, H. and Castro, F (2014) A morphometric and molecular approach to define three closely related species of frogs of the Genus *Pristimantis* (Anura: Craugastoridae) from the Cordillera Occidental in Colombia. Journal of Herpetology, 48(2): 220–227.

Gonzalez-Duran, G.A., Targino, M., Rada, M. and Grant, T (2017) Phylogenetic relationships and morphology of the *Pristimantis leptolophus* species group (Amphibia: Anura: Brachycephaloidea), with the recognition of a new species group in *Pristimantis* Jiménez de la Espada, 1870. Zootaxa, 4243(1): 42–74.

Hedges SB, Duellman WE, Heinicke MP (2008) New World direct-developing frogs (Anura: Terrarana): molecular phylogeny, classification, biogeography, and conservation. Zootaxa 1737: 1–182. https://doi.org/10.11646/zootaxa.1737.1.1

Heinicke, M. P., W. E. Duellman, and S. B. Hedges (2007) Major Caribbean and Central American frog faunas originated by ancient oceanic dispersal. Proceedings of the National Academy of Sciences of the United States of America 104: 10092–10097.

Köhler J, Padial JM (2016) Description and phylogenetic position of a new (singleton) species of *Oreobates* Jiménez de la Espada, 1872 (Anura: Craugastoridae) from the Yungas of Cochabamba, Bolivia. Annals of the Carnegie Museum. Pittsburgh 84: 23–38. https://doi.org/10.2992/007.084.0104

Lehr, E., Fritzsch, G. and Müller, A (2005) Analysis of Andes frogs (*Phrynopus*, Leptodactylidae, Anura) phylogeny based on 12S and 16S mitochondrial rDNA sequences. Zoologica Scripta, 34(6): 593–603.

Lehr, E., von May, R., Moravec, J. and Cusi, J.C (2017) A new species of *Phrynopus* (Amphibia, Anura, Craugastoridae) from upper montane forests and high Andean grasslands of the Pui Pui Protected Forest in central Peru. ZooKeys, (713): 131.

Mendoza, Á.M., Ospina, O.E., Cárdenas-Henao, H. and García-R, J.C (2015) A likelihood inference of historical biogeography in the world’s most diverse terrestrial vertebrate genus: Diversification of direct-developing frogs (Craugastoridae: *Pristimantis*) across the Neotropics. Molecular phylogenetics and evolution, 85: 50–58.

Motta AP, Chaparro Auza JC, Pombal JP, Guayasamin JM, De la Riva I, Padial JM (2016) Molecular phylogenetics and taxonomy of the Andean genus *Lynchius* Hedges, Duellman, and Heinicke, 2008 (Anura: Craugastoridae). Herpetological Monographs 30: 119–142. https://doi.org/10.1655/HERPMONOGRAPHS-D-16-00002

Motta, A. P., P. P. G. Taucce, C. F. B. Haddad, and C. Canedo (2021) A new terraranan genus from the Brazilian Atlantic Forest with comments on the systematics of Brachycephaloidea (Amphibia: Anura). Journal of Zoological Systematics and Evolutionary Research 59: 663–679. http://doi.org//10.1111/jzs.12452

Ortega-Andrade, H.M., Rojas-Soto, O.R., Valencia, J.H., Espinosa de los Monteros, A., Morrone, J.J., Ron, S.R. and Cannatella, D.C (2015) Insights from integrative systematics reveal cryptic diversity in *Pristimantis* frogs (Anura: Craugastoridae) from the Upper Amazon Basin. PloS one, 10(11), p.e0143392.

Ortega JA, Cisneros-Heredia DF, Camper JD, Romero-Carvajal A, Negrete L, Ron SR (2025) Systematics of minute strabomantid frogs allocated to the genus *Noblella* (Amphibia: Anura) with description of a new genus, seven new species, and insights into historical biogeography. Zoological Journal of the Linnean Society 203: 1–60. https://doi.org/10.1093/zoolinnean/zlae162

Padial JM, Chaparro JC, I. De la Riva (2008) Systematics of *Oreobates* and the *Eleutherodactylus discoidalis* species group (Amphibia, Anura), based on two mitochondrial DNA genes and external morphology. Zoological Journal of the Linnean Society 152: 737–773. https://doi.org/10.1111/j.1096-3642.2007.00372.x

Padial, J. M., S. Castroviejo-Fisher, and I. De la Riva (2009a) The phylogenetic relationships of *Yunganastes* revisited (Anura: Terrarana). Molecular Phylogenetics and Evolution 52: 911–915.

Padial, J.M. and De la Riva, I (2009b) Integrative taxonomy reveals cryptic Amazonian species of *Pristimantis* (Anura: Strabomantidae). Zoological Journal of the Linnean Society, 155(1), pp.97–122.

Padial JM, Chaparro Auza JC, Castroviejo-Fisher S, Guayasamin JM, Lehr E, Delgado AJ, Vaira M, Teixeira M, Aguayo-Vedia CR, De la Riva I (2012) A revision of species diversity in the Neotropical genus *Oreobates* (Anura: Strabomantidae), with the description of three new species from the Amazonian slopes of the Andes. American Museum Novitates 3752: 1–55 http://digitallibrary.amnh.org/handle/2246/6321

Pereyra, M. O., D. E. Cardozo, J. L. Baldo, and D. Baldo (2014) Description and phylogenetic position of a new species of *Oreobates* (Anura: Craugastoridae) from northwestern Argentina. Herpetologica 70: 211–227.

Reyes-Puig, C., Yánez-Muñoz, M.H., Ortega, J.A. and Ron, S.R (2020) Relaciones filogenéticas del subgénero Hypodictyon (Anura: Strabomantidae: *Pristimantis*) con la descripción de tres especies nuevas de la región del Chocó. Revista mexicana de biodiversidad, 91.

Reyes-Puig, J.P., Reyes-Puig, C., Ron, S., Ortega, J.A., Guayasamin, J.M., Goodrum, M., Recalde, F., Vieira, J.J., Koch, C. and Yánez-Muñoz, M.H (2019) A new species of terrestrial frog of the genus *Noblella* Barbour, 1930 (Amphibia: Strabomantidae) from the Llanganates-Sangay Ecological Corridor, Tungurahua, Ecuador. PeerJ, 7, p.e7405.

Reyes-Puig, C., Maynard, R.J., Trageser, S.J., Vieira, J., Hamilton, P.S., Lynch, R., Culebras, J., Kohn, S., Brito, J. and Guayasamin, J.M (2020) A new species of *Noblella* (Amphibia: Strabomantidae) from the Río Manduriacu Reserve on the Pacific slopes of the Ecuadorian Andes. Neotropical Biodiversity, 6(1):162–171.

Reyes-Puig, C., Guayasamin, J.M., Koch, C., Brito-Zapata, D., Hollanders, M., Costales, M. and Cisneros-Heredia, D.F (2021) A new species of the genus *Noblella* (Amphibia: Strabomantidae) from Ecuador, with new information for *Noblella worleyae*. Acta Herpetologica, 16(2): 63–87.

Reyes-Puig JP, Yánez-Muñoz MH, Urgilés-Merchán MA, Libke Z, Ron SR, Carrión-Olmedo JC (2026) Two new terrestrial narrow-toed frogs (*Niceforonia*: Strabomantidae, Anura) from remote unexplored mountains in the Ecuadorian Andes. PeerJ 14(e21182): 1–30. https://doi.org/10.7717/peerj.21182

Sánchez-Nivicela, J. C., V. L. Urgilés, M. J. Navarrete Méndez, M. H. Yánez-Muñoz, and S. R. Ron (2019) A bizarre new species of *Lynchius* (Amphibia, Anura, Strabomantidae) from the Andes of Ecuador and first report of *Lynchius parkeri* in Ecuador. Zootaxa 4567: 1–24. https://doi.org/10.11646/zootaxa.4567.1.1

Santa-Cruz Farfan R, von May R, Catenazzi A, Whitcher C, López Tejeda E, Rabosky DL (2019) A new species of terrestrial-breeding frog (Amphibia, Strabomantidae, *Noblella*) from the Upper Madre De Dios watershed, Amazonian Andes and lowlands of southern Peru. Diversity 11 (145): 1–20. https://doi.org/10.3390/d11090145

Vaz-Silva, W., N. M. Maciel, S. P. de Andrade, and R. C. Amaro (2018) A new cryptic species of *Oreobates* (Anura: Craugastoridae) from the seasonally dry tropical forest of central Brazil. Zootaxa 4441: 89–108. https://doi.org/10.11646/zootaxa.4441.1.5

Venegas, P.J., Barboza, A.C., De la Riva, I. and Padial, J.M (2018) A new species of *Phrynopus* from the northeastern Andes of Peru, its phylogenetic position, and notes on the relationships of Holoadeninae (Anura: Craugastoridae). Zootaxa, 4446(4): 501–524.

von May R, Catenazzi A, Corl A, Santa-Cruz R, Carnaval AC, Moritz C (2017) Divergence of thermal physiological traits in terrestrial breeding frogs along a tropical elevational gradient. Ecol Evol. 7: 3257–3267. https://doi.org/10.1002/ece3.2929

von May, R., Lehr, E. and Rabosky, D.L (2018) Evolutionary radiation of earless frogs in the Andes: molecular phylogenetics and habitat shifts in high-elevation terrestrial breeding frogs. PeerJ, 6, p.e4313.

von May R, Diaz MI, Ttito A, anta-Cruz Farfan R, Catenazzi A (2024) The rediscovery of *Noblella peruviana* after more than 115 years helps resolve the molecular phylogeny and taxonomy of Noblella (Amphibia, Anura, Strabomantidae). Diversity 16 (613): 1–15 https://doi.org/10.3390/d16100613

Zhang, P., Liang, D., Mao, R.L., Hillis, D.M., Wake, D.B. and Cannatella, D.C (2013) Efficient sequencing of anuran mtDNAs and a mitogenomic exploration of the phylogeny and evolution of frogs. Molecular Biology and Evolution, 30(8): 1899–1915.
